# Telehealth-Delivered Program and Accompanying Patients to Enhance the Clinical Condition of Patients Throughout a Liver Transplant: Protocol for a Mixed Methods Study

**DOI:** 10.2196/54440

**Published:** 2024-03-22

**Authors:** Marie-Pascale Pomey, Enora Le Roux, Nathalie Nadon, Jessie Perron, Angèle Barry, Chantal Bémeur, Thomas G Poder, Fernand Duford, Louise Laviolette, Johanne Tétrault-Lassonde, Cécile Vialaron, Manuel J Escalona, Louise Normandin, Geneviève Huard, Catherine Girardin, Christopher Rose, Kathy Malas, Denis Ouellet, Catherine Vincent

**Affiliations:** 1 Carrefour de l’Innovation Centre de recherche du Centre hospitalier de l'Université de Montréal Montréal, QC Canada; 2 Département de gestion évaluation et politique de santé, école de santé publique Université de Montréal Montreal, QC Canada; 3 Centre d’excellence sur le partenariat avec les patients et le public Montreal, QC Canada; 4 Université Paris Cité ECEVE UMR 1123, Inserm, Faculté de Médecine Paris France; 5 AP-HP.Nord-Université Paris Cité Hôpital Universitaire Robert Debré Unité d'épidémiologie clinique, Inserm, CIC 1426 Paris France; 6 Regroupement cardio-vasculaire, hépatologie et transplantation Centre hospitalier de l'Université de Montréal Montreal, QC Canada; 7 Axe immunopathologie Centre de recherche du Centre hospitalier de l'Université de Montréal Montreal, QC Canada; 8 Réseau transplantation et cliniques externes de transplantation et d’hépatologie Centre hospitalier de l'Université de Montréal Montreal, QC Canada; 9 Axe Cardiométabolique Centre de recherche du Centre hospitalier de l'Université de Montréal Montreal, QC Canada; 10 Centre de recherche de l’IUSMM CIUSSS de l’Est de l’Île de Montréal Montreal, QC Canada; 11 Département d’hépatologie Centre hospitalier universitaire de Montréal (CHUM) Montreal, QC Canada; 12 Direction de la biovigilance et de la biologie médicale Ministère de la Santé et des Services sociaux du Québec Montreal, QC Canada

**Keywords:** liver transplantation, accompanying patients, connected objects, health care model, digital platform

## Abstract

**Background:**

Liver transplantation (LT) is indicated in patients with severe acute or chronic liver failure for which no other therapy is available. With the increasing number of LTs in recent years, liver centers worldwide must manage their patients according to their clinical situation and the expected waiting time for transplantation. The LT clinic at the Centre hospitalier de l’Université de Montréal (CHUM) is developing a new health care model across the entire continuum of pre-, peri-, and posttransplant care that features patient monitoring by an interdisciplinary team, including an accompanying patient; a digital platform to host a clinical plan; a learning program; and data collection from connected objects.

**Objective:**

This study aims to (1) evaluate the outcomes following the implementation of a patient platform with connected devices and an accompanying patient, (2) identify implementation barriers and facilitators, (3) describe service outcomes in terms of health outcomes and the rates and nature of contact with the accompanying patient, (4) describe patient outcomes, and (5) assess the intervention’s cost-effectiveness.

**Methods:**

Six types of participants will be included in the study: (1) patients who received transplants and reached 1 year after transplantation before September 2023 (historical cohort or control group), (2) patients who will receive an LT between December 2023 and November 2024 (prospective cohort/intervention group), (3) relatives of those patients, (4) accompanying patients who have received an LT and are interested in supporting patients who will receive an LT, (5) health care professionals, and (6) decision makers. To describe the study sample and collect data to achieve all the objectives, a series of validated questionnaires, accompanying patient logbooks, transcripts of interviews and focus groups, and clinical indicators will be collected throughout the study.

**Results:**

In total, 5 (steering, education, clinical-technological, nurse prescription, and accompanying patient) working committees have been established for the study. Recruitment of patients is expected to start in November 2023. All questionnaires and technological platforms have been prepared, and the clinicians, stakeholders, and accompanying patient personnel have been recruited.

**Conclusions:**

The implementation of this model in the trajectory of LT recipients at the CHUM may allow for better monitoring and health of patients undergoing transplantation, ultimately reducing the average length of hospital stay and promoting better use of medical resources. In the event of positive results, this model could be transposed to all transplant units at the CHUM and across Quebec (potentially affecting 888 patients per year) but could also be applied more widely to the monitoring of patients with other chronic diseases. The lessons learned from this project will be shared with decision makers and will serve as a model for other initiatives involving accompanying patients, connected objects, or digital platforms.

**International Registered Report Identifier (IRRID):**

PRR1-10.2196/54440

## Introduction

### Background

Liver transplantation (LT) is a surgical procedure to remove a diseased or injured liver that results in liver failure and replaces it with a healthy liver from a donor. LT is indicated in patients with severe acute or chronic liver failure for which no other therapy is available or with hepatocellular carcinoma. Acute liver failure has several causes, including viral (most commonly hepatitis B and C) and drug-induced hepatitis. Chronic liver failure can also have several causes, such as alcoholic liver disease, autoimmune liver disease, and fatty liver disease. LT offers an opportunity to improve not only the health-related quality of life but also the life span of patients living with chronic liver disease and its associated complications.

Liver failure is now the seventh leading cause of death worldwide, with 1.4 million deaths per year. In Europe, >5000 LTs are performed per year, with >140,000 LTs performed over the past 6 years [1] for a European population of 751 million. In the United States, 8906 LTs were performed in 2020 alone, with a total of 24,936 candidates listed for LTs that same year [2]. In Canada, liver failure is the 12th leading cause of death, particularly among the population aged between 25 and 64 years. Cumulative LT activity in Canada has reached 27,488 patients, including 498 transplants from living donors since 1998. In 2020, a total of 565 LTs were reported, with the transplantation rate increasing by 22% over the past 10 years [3]. However, the growing need for LTs also increases the number of candidates on the waiting list, which is greater than the number of viable livers. As a result, the waiting time for transplantations can vary from a few days to >1 year depending on the patient’s condition [1].

With the growing number of LTs, liver centers worldwide must manage their patients according to their clinical situation and the expected waiting time for transplants. Moreover, the LT procedure and postoperative period adversely affect the patient’s well-being. Therefore, patients should be carefully prepared both physically and mentally to undergo transplantation. Proper management of patients on the waiting list is essential to avoid death or dropout because of deterioration of their condition, as well as to ensure that patients are in the best possible physical, psychological, and social condition before the procedure. These factors are key to the success of the postoperative course [1]. Moreover, the organization of outpatient care is characterized by a certain number of dysfunctions, such as numerous medical consultations with several specialists, which require that patients travel several times to appointments. This is particularly the case with pretransplant assessments, for which patients may need to meet with numerous specialists and undergo several biological, clinical, and imaging tests. These numerous trips put a heavy burden on patients, often resulting in outdated examination results such that patients then have to retake them, unnecessary consultations, and a significant mobilization of caregivers.

However, implementing a model that will meet these requirements is costly and requires the availability of all qualified professionals involved in the LT process. Given the limited human resources in care services, follow-up tools such as mobile apps and web-based platforms may strengthen patient adherence and help empower patients who have undergone transplantation [4]. The mobilization of patients who have undergone transplantation or former patients to provide additional support may also improve patients’ motivation and engagement in care [5]. Finally, involving professionals with a mission of health promotion may have an impact on the immediate and long-term health of patients with liver disease. Given this context, the University of Montréal Health Center (*Centre hospitalier de l’Université de Montréal*; CHUM) has decided to implement an innovative health care model to speed up pretransplant assessments, provide interdisciplinary support, and implement social and technological innovations that will enhance patient health.

### Aims and Objectives

Drawing on implementation science, this mixed methods pilot study will evaluate the implementation of a new health care model to enhance the clinical condition of patients throughout their LT experience. In line with the outcome categories proposed for implementation research [6], this study’s specific objectives are to (1) quantitatively evaluate the implementation outcomes of a patient platform through connected devices and accompaniment by a former patient (in terms of predefined benchmarks for acceptability, usability, response burden, feasibility [recruitment and retention], and fidelity), (2) identify implementation barriers and facilitators through semistructured interviews with stakeholders, (3) describe service outcomes in terms of health outcomes and the rates and nature of contact with the accompanying patient, (4) describe patient outcomes (based on daily self-reported health data, including symptoms, use of health services beyond the intervention, and patient satisfaction with teleconsultations if received), and (5) assess the cost-effectiveness of the intervention.

### Intervention Implementation Strategy

#### Overview of the Strategy

This study draws on implementation sciences [7] that are intended to foster the adoption or implementation of an intervention. The selected strategies are presented based on the main corresponding implementation phase: preimplementation, implementation, and postimplementation phase. Central to our implementation strategy is our intention to involve patients and stakeholders through various engagement approaches. Our stakeholder engagement strategy is implemented by an interdisciplinary research team that includes not only researchers (MPP, ELR, CB, TP, CV, CR, and MJE), clinicians (CV, AB, GH, and CG), managers (NN and JP), and decision makers (KM and DO) but also patient coresearchers and former patients (FD, LL, and JTL) [8-11]. Our research and intervention development processes are based on a coconstruction methodology that respects the knowledge and responsibility of the stakeholders based on their values, expertise, and perspectives concerning a health condition and its associated care [12,13]. By involving all stakeholders in this project from the outset, we seek not only to detect and address the challenges associated with recruitment, accessibility, acceptability, and the comprehensibility of procedures and instruments [14] but also to enhance the relevance and meaningfulness of our research results [12].

At the CHUM, certain sectors of activity have been prioritized, including the LT clinic, to improve the patient pathway. The LT clinic constitutes a great target for work on a new model of outpatient care as the medical and nonmedical teams are highly motivated; the number of patients is manageable (60 patients undergoing transplants per year); and the clinic offers a platform where it is possible to integrate and coordinate several medical specialties, such as hepatology, nephrology, cardiology, endocrinology, and even psychiatry.

#### Overview of the Intervention

The proposed intervention is complex; it “comprises multiple interacting components, although additional dimensions of complexity include the difficulty of their implementation and the number of organizational levels they target” [15]. This new health care model is called Transplant’Action Connected in Liver Transplant (TAC) and includes five components implemented before, during, and after LT: (1) clinical team and technical support; (2) a kinesiology-based intervention plan; (3) a nutritional intervention plan; (4) peer support; and (5) the use of a digital platform, including an interface containing the intervention plans with support videos as well as the association of connected objects (COs; ie, a blood pressure monitor, scale, bracelet, and glucometer [if necessary]) to monitor patients’ biological variables and physical activity.

##### Before Transplantation

In the regular care at the CHUM, patients who could potentially benefit from a transplant are assessed by the CHUM’s *guichet rapide d’investigation en transplantation du foie* (rapid LT assessment service; GRIT-F), which provides a 1-week pretransplant assessment on an outpatient basis. GRIT-F usually includes a clinical team composed of hepatologists, a pretransplant nurse, and a cardiologist if necessary. Following this assessment, if the results are favorable, the patient is placed on the transplant waiting list. The patient is then monitored exclusively by the hepatologist every 3 months (or more often if the patient presents signs of deterioration). No systematic follow-up by a nurse or other health care professionals (HCPs) is provided during this period.

In the new TAC health care model, the GRIT-F will include additional professionals, such as a kinesiologist, nutritionist, psychologist, cardiologist (if needed), social worker, and accompanying patients. This team assesses not only the patient’s clinical situation but also the patient’s knowledge and understanding of the disease, living with the disease, the transplant, and drug treatments, as well as their digital literacy. If the patient is placed on the transplant list, the patient benefits from a 1-day individual meeting with the CHUM pretransplant team, consisting of the clinical team (hepatologist, nurse, nutritionist, kinesiologist, and accompanying patient) and the digital health specialist. During this day, an intervention plan is coconstructed with the patient that includes activities to be carried out as well as the fitness and nutritional goals to be achieved. To support them in implementing this intervention plan, the patient receives COs (ie, a blood pressure monitor, scale, bracelet to measure physical activity, and glucometer) as well as access to a digital platform that provides physical exercises and nutritional recommendations. This digital platform tracks the data collected by the COs and provides access to videos. A support plan is also implemented. It includes a follow-up by the transplant nurse every 4 weeks via teleconsultation, by the accompanying patient every month, and by the nutritionist and kinesiologist every month for the first 2 months and upon request thereafter. Technological support is also provided throughout this phase.

##### During Transplantation

In the regular care provided at the CHUM, the patient is followed by the medical team and the transplant floor nurse and is seen by the pharmacist for medication education. In addition, the services of a physiotherapist and a nutritionist are available on the transplant floor.

In the new TAC health care model, other professionals, such as psychologists, kinesiologists, and nutritionists, will intervene according to the patient’s needs. However, the patient who undergoes transplantation continues to benefit from the support of an accompanying patient. Depending on their clinical condition, the patient can perform kinesiology exercises using a bicycle adapted to the bed. The use of COs during this phase is left to the patient’s discretion.

##### After Transplantation

As part of the regular care at the CHUM, the patient is followed only by the hepatologist and posttransplant follow-up nurse in the months following the transplant.

**Figure 1:**
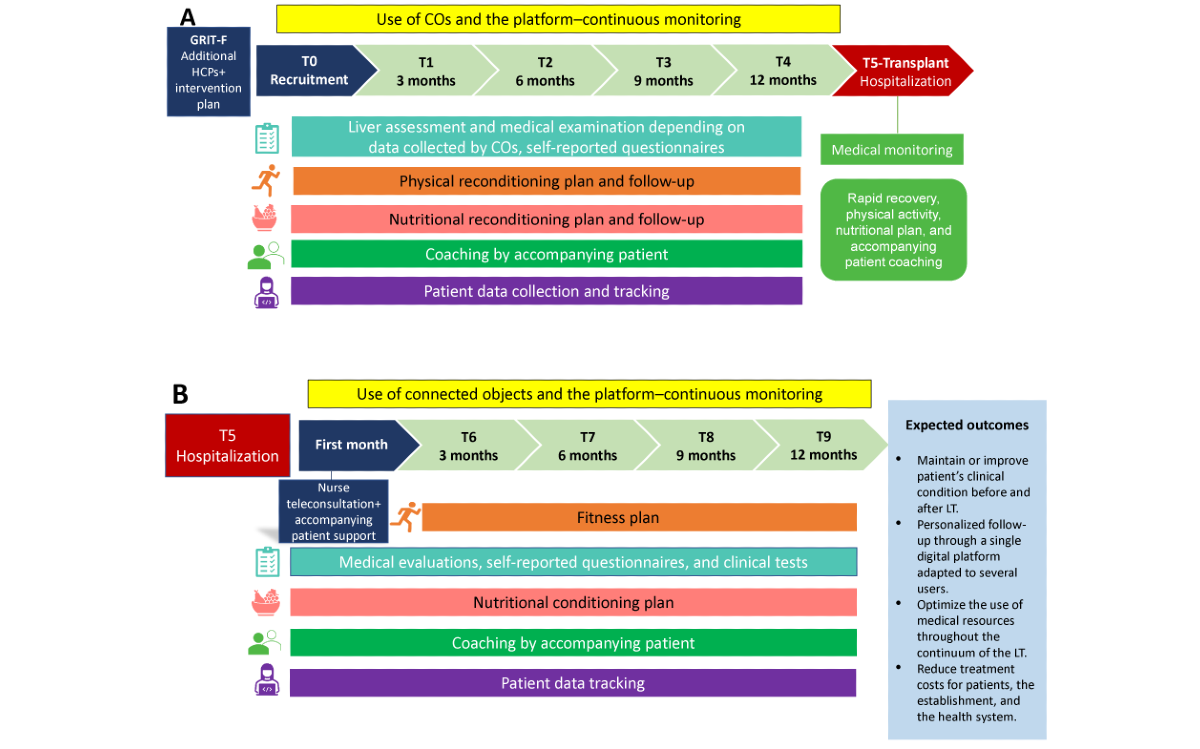
Overall view of the intervention indicating key time points for data collection during (A) the pretransplant period and (B) the posttransplant period. CO: connected object; GRIT-F: guichet rapide d'investigation en transplantation du foie (rapid liver transplant assessment service); HCP: health care professional; LT: liver transplant.

As part of the new TAC health care model, upon returning home, the patient continues to receive weekly support via videoconference from the nurse and the accompanying patient during the first month and then monthly for 1 year, as well as in-person meetings with the clinical team. The patient resumes the use of COs at any time, and the nutritional plan is adapted as needs emerge. When allowed by the patient’s general condition (eg, healing and muscular strength), a new physical exercise plan is proposed in the first 3 to 6 months after transplantation and carried out under the supervision of the kinesiologist and accompanying patient. An overview of the TAC health care model is presented in Figure 1.

#### Strategies During the Implementation Phase

All the HCPs and accompanying patients will be trained in therapeutic education and motivational interviewing to support the patients throughout their treatment, as well as in the use of the digital platform and COs. The team’s role is to provide recommendations on the use of the digital platform, the procedures to follow as part of their intervention plan, the procedures for remote monitoring using the COs, the patient’s daily self-assessment, and information on the educational material to be provided to participants through the patient portal.

When patients receive their COs, they will be supported by the digital platform developers and by the CHUM telehealth coordination center. To centralize technical support, the CHUM telehealth coordinator will communicate with the digital platform development team (ML and ECN) to maintain, configure, and update the patient portal for the duration of the project. Regular meetings will be held to discuss the configuration of the digital platform to ensure that it integrates stakeholder recommendations as obtained from engaged HCPs, accompanying patients, and patients. RdP will also be available to provide technical assistance to patients via email throughout the study.

To ensure that the patients’ needs are met, the accompanying patients and HCPs will be regularly consulted to obtain their feedback on the proposed procedures for remote monitoring and research material (eg, informed consent documents and all the data collection instruments, including the self-assessment). This will help ensure the relevance, comprehensibility, and comprehensiveness of the results.

To effect cyclical change, MPP is planning 4 prototype tests with engaged HCPs (ie, the clinical team), accompanying patients, and a sample of patients. For each test, the members will receive training on the digital platform; use it for 4 days; and then meet to discuss their experiences, report any problems, and make recommendations. Furthermore, a test is planned of the patient app portal involving 5 participants to adjust the intervention as required before the project begins with the patients. During that time, all HCPs, accompanying patients, and researchers will be trained on how to use the platform.

Once the platform is ready, patients will be registered to use it. Participants will be guided by a member of the research staff (CV or ME), who will confirm their identity and explain platform functionalities. The remote registration system will send a unique verification code to the user’s smartphone with a link to the registration page, where the code will be entered. This is to ensure the security and privacy of patient information.

#### Strategy During the Postimplementation Phase

Our strategy is to re-examine the implementation phase by analyzing the participants’ and stakeholders’ views on implementation barriers and facilitators after implementation (based on qualitative interviews).

## Methods

### Study Design

A single-center pilot study will be conducted following the guidance provided by the CONSORT (Consolidated Standards of Reporting Trials) statement for pilot and feasibility studies [16,17]. It uses a mixed methods embedded design, with quantitative methods to measure the outcome and output and qualitative methods to extend the analysis of the implementation process [18].

### Participants

To attain the study objectives, six types of participants will be included in the study: (1) patients who underwent transplantation 2 years before and received regular care (historical cohort or control group); (2) patients who will receive an LT during the next year under the new health care model (prospective cohort/intervention group); (3) relatives of the patients who will receive an LT; (4) accompanying patients who have already received an LT and are interested in supporting patients who will receive an LT; (5) HCPs, including proximity managers; and (6) decision makers.

#### Eligibility Criteria

##### Patient Population

All patients selected for an LT at the CHUM who are aged >18 years at the time of registration on the transplant waiting list and are fluent in French or English (written and spoken) will be considered for participation in the study. In addition, patients in the prospective cohort who will receive an LT will have to be able to use COs and remote monitoring platforms. The exclusion criterion for persons in the intervention group is not having a relative who can provide support in their use of the platform and the COs.

##### Other Population

Persons aged >18 years who are fluent in French or English (written and spoken) and linked to the study will be considered for participation.

#### Recruitment and Sample

##### Patient Population (Control Group)

This comprises patients *not exposed* to the new health care model. It consists of a historical cohort focused on the last 50 patients who underwent transplantation and reached 1 year after transplantation before September 2023. They will be identified through the hospital archive department.

##### Patient and Relative Population (Intervention Group)

This comprises patients (and their relatives) who will receive an LT from December 2023 to November 2024. Knowing that 60 patients receive an LT at the CHUM each year and that it is expected that 80% of these patients will be selected for the research project, we expect that 48 patients will be enrolled. This percentage is an estimate made by the clinical team based on their knowledge of the target population. During the week of the GRIT-F review, the research project will be presented to patients by the accompanying patient and the nurse. On the day that the patient is listed to receive an LT, the research assistant (CV or ME) will meet with the patient to present the project, give the informed consent form, answer their questions, and collect their consent or refusal to participate in the study. Patients who refuse to participate in the study will have the team’s support without access to the digital platform and will not have any data collected in addition to what is routinely collected at the clinic.

##### Accompanying Patients and HCPs

This includes all the persons in these categories involved in patient care.

##### Decision Makers

This includes all persons involved in facilitating the implementation of the new health care model. It comprises the chairman and chief executive officer, the deputy chairman and chief executive officer, the director of medical affairs or their representative, the director of nursing or their representative, the director of multidisciplinary care or their representative, the director of the Information Technologies and Telecommunications Department or their representative, the director of the Technological Integration and Interoperability Operationalization Centers Department or their representative, the director of the Network Coordination Department or their representative, and the director of research or their representative.

### Procedures

Table 1 presents all the study procedures and clinical and output indicators for patient data collection and evaluation to be used in the study. Table 2 details the data collection procedures to be used for accompanying patients, HCPs, and decision makers throughout the study.

**Table 1:** Clinical and output indicators for patient data collection and evaluation.

Data source and description		Collection time
**Clinical examinations**		
	Hepatic encephalopathy using the Stroop test	Every 3 months (during the medical visit)
	Liver Frailty Index	Every 3 months (during the medical visit)
	6MWT^a^	During the personal meeting day
	Calf circumference	During the personal meeting day
	Clavien-Dindo score (eg, presence of infectious complications, presence of wound dehiscence, and revision surgery) [1]	At the end of hospitalization
**COs^b^**		
	Blood pressure	3 times a week
	Weight	3 times a week
	Capillary glucose (if needed)	3 times a week
	Pulse	3 times a week
	Number of steps and type of exercise	Continuously (ie, 3 times per day, 7 days per week)
	Number of false alerts	Continuously
	Number of interventions to solve problems related to COs	Continuously
	Replacement of COs because of technological problems	Continuously
**Medical records**		
	Number of medical visits (hepatologist, nephrologist, cardiologist, endocrinologist, psychiatrist, medical specialist in addiction, and dentist; via telephone or in person)	Continuously
	Number of nonmedical visits (via telephone or in person)	Continuously
	Number of emergency room visits	Continuously
	Number of hospitalization days while waiting for the transplant and the reasons for these hospitalizations	Continuously
	Number of days between registration on the transplant list and transplant	Continuously
	Average length of stay (hospitalization days) for the transplant	At the end of hospitalization
	Number of rehospitalizations in the first year after the transplant (average duration, location [eg, intensive care], and reasons for these hospitalizations)	Continuously
	Surgical revision	Continuously

^a^6MWT: 6-Minute Walk Test.

^b^CO: connected object.

**Table 2:** Data collection and procedures for accompanying patients, health care professionals, and decision makers throughout the study.

Data source		Description	Collection time
**Accompanying patients**			
	Documents	Logbook	Continuously
	Questionnaires	Compassion fatigue risk test [19]	At the beginning and end of the study
	Focus group or interview	Perception of the contribution of the new health care model to follow-up and user experience	At the beginning and end of the study
**Health care professionals**			
	Questionnaires and focus group	Workplace Ethical Climate Scale [20] Ability to work with accompanying patients [21] Contributions of this model of care to improving the patient journey and appropriate use of specialized medical resources+evaluation of technical difficulties and the user-friendliness of the data accessed from medical records	At the beginning and end of the study
**Decision makers**			
	Interview	Contributions of this model of care and its limits and impact on the service	At the beginning and end of the study

### Study Data Collection and Metrics

#### Platform and Connected Device Implementation Questionnaires

To assess the implementation outcomes (for objective 1), a series of interviews will be conducted 1 and 6 months before the transplant and 6 and 12 months after the transplant. These interviews will include questions on acceptability, response burden, and usability.

To evaluate the effort required to complete the questionnaires and further evaluate acceptability, the perceived response burden measure [22] will be used. It consists of a single question on a 5-point response scale adapted from a survey question from the UK Office for National Statistics.

Usability (the extent to which users can achieve the specific goal of a product) and acceptability (how agreeable, pleasant, or satisfactory the intervention is to the stakeholders [6]) are considered important aspects of implementation outcomes [23]. Both aspects will be assessed using the System Usability Scale questionnaire [24], which consists of 10 items rated on a 5-point Likert scale that are then averaged to produce a total score from 1 to 5. The participants’ scores for each question are converted to a new number, added together, and then multiplied by 2.5 to convert the original scores from a scale from 0 to 40 to a scale from 0 to 100. A System Usability Scale score of >68 will indicate that the technology is considered to have an important impact and be useful and easy to use.

Satisfaction with teleconsultations (for objective 4) will be evaluated using the Telemedicine Satisfaction Questionnaire [25], which will be sent to patients after each teleconsultation.

#### Clinical Questionnaires

To describe the study sample and collect the data needed to achieve the study objectives, a series of validated questionnaires will be completed by all patients. The questionnaires will cover patient characteristics, the assessment of the partnership relationship between patients and professionals (CADICEE) [26], quality of life (SF-6Dv2) [27], the ability to engage in one’s own care (PAM) [28], the perception of one’s ability to change one’s lifestyle habits [29], monitoring of compliance with medication treatments [30], internet-based 24-hour recall (dietary assessment) [31], the Subjective Global Assessment [32], out-of-pocket costs by patients for health care (CoPaQ) [33], and medical visits and consultations. Table 3 presents a summary of the timing and frequency of data collection. The secure internet-based platform REDCap (Research Electronic Data Capture; Vanderbilt University), an application for building and managing web-based surveys and databases, will be used to administer the questionnaires, organize the data collection, and analyze the data.

**Table 3:** Validated questionnaires for patient data collection and evaluation.

Domain and questionnaire		Dimensions	Number of items	Response scale	Time of measurement
**Patient information**					
	Sociodemographic, medical, and capacity for use of COs^a^ questionnaire	Gender, age group, highest level of education, marital status, native language, ethnicity, and employment status Medical background, smoking habits, other medical conditions, and chronic diseases (other than chronic liver disease) Use of COs during a research study in the past	11 items	Varied	T0^b^
**Emotional evaluation**					
	CADICEE questionnaire	Evaluates the foundations of the partnership relationship between patients and health care professionals Identifies potential gaps in the partnership	27 items	Varied	T0, T2^c^, hospitalization, T7^d^, and T9^e^
	SF-6Dv2 Health Utility Survey	Measures of health for QALY^f^ calculations Provides keen insights into patient healing by measuring 6 health domains: physical functioning, role limitations, social functioning, pain, mental health, and vitality	7 items	5-point Likert scale (1=not at all; 5=nearly all the time)	T0, T4^g^, T6^h^, and T9
**Treatment monitoring and lifestyle habits**					
	PAM-13	Ascertains the patient’s self-reported knowledge, skills, and confidence in the self-management of their health	13 items	4-point Likert scale (1=strongly disagree; 4=strongly agree)	T0, T4, T6, and T9
	Monitoring compliance with medication treatments	Explores the intention or the need for help with current treatment plans	8 items	5-point Likert scale (1=strongly disagree; 5=strongly agree)	T0, T1^i^, T2, T4, T6, T7, T8^j^, and T9
**Financial monitoring**					
	Out-of-pocket costs of health care for patients (CoPaQ)	Collects net costs related to the patient’s state of health that are not reimbursed by their insurers	33 items	Varied	T0, T1, T2, T4, T6, T7, T8, and T9
**Clinical and health condition monitoring**					
	Online 24-hour recall	Retrospective method that monitors and assesses an individual’s food and drink consumption during the previous day	Electronic interface (images)	Varied	T0, T1, T2, T4, T6, T7, T8, and T9
	SGA^k^	Diagnose malnutrition and identify those who would benefit from nutrition care History of recent intake, weight change, gastrointestinal symptoms, and a clinical evaluation	Clinical assessment	Varied	T0, T1, T2, T4, T6, T7, T8, and T9
	Medical visits and consultations	Continuous monitoring of medical visits or consultations and the reasons for them	Continuous medical monitoring	Varied	T0, T1, T2, T4, T6, T7, T8, and T9
**Hospital care**					
	Quality of care in hospitalization scale (ESQ-H)	Self-reported questionnaire with 2 domains measuring patient satisfaction with the quality of medical and nursing care in hospitals It contains 2 domains: quality of medical information and the relationship with staff and daily routine	18 items	5-point Likert scale (1=poor; 5=excellent)	Hospitalization

^a^CO: connected object.

^b^T0: recruitment.

^c^T2: 6 months after recruitment.

^d^T7: 6 months after transplantation.

^e^T9: 12 months after transplantation.

^f^QALY: quality-adjusted life year.

^g^T4: 12 months after recruitment.

^h^T6: 3 months after transplantation.

^i^T1: 3 months after recruitment.

^j^T8: 9 months after transplantation.

^k^SGA: Subjective Global Assessment.

#### Clinical Indicators

Clinical indicators will be determined by the physician in charge of the patient or the team’s kinesiologist. Among the clinical tests specific to this study, the physician will explore signs of hepatic encephalopathy (ie, deterioration of brain function that occurs in people with severe liver failure) using the Stroop test [34] to assess psychomotor speed and cognitive flexibility by recording the response time to interference between recognizing color fields and writing color names performed at different time points during the study (Table 3). Other clinical tests include the Liver Frailty Index [35], which consists of 3 tests representing 3 major components of the multidimensional construct of frailty in patients with cirrhosis: grip strength, chair stands (muscle weakness), and balance (altered neuromotor coordination). Moreover, the 6-Minute Walk Test (6MWT) [36], which assesses aerobic capacity and endurance, will be performed by the team’s kinesiologist during the initial patient assessment (baseline) and every 3 months after the LT. The 6MWT measures the distance covered in 6 minutes (outcome) and will be used to identify changes in performance capacity. In conjunction with the 6MWT, the kinesiologist will also measure calf circumference [37], a representative anthropometric index used to screen for sarcopenia and in patient follow-up to adapt nutritional and exercise plans to meet each patient’s needs throughout the posttransplant period. Other clinical indicators will be collected using COs: blood pressure, weight, pulse, number of steps/type of exercise, and capillary glucose (if needed).

In addition, to monitor the platform’s reliability, we will look at the number of false alerts, the number of interventions required to solve problems related to COs, and the replacement of COs because of technological problems.

Output indicators will also be collected: number of medical visits (hepatologist, nephrologist, cardiologist, endocrinologist, psychiatrist, medical specialist in addiction, and dentist; via telephone, on the platform, or in person), number of nonmedical visits (via telephone, on the platform, or in person), number of emergency room visits, number of hospitalization days while waiting for the transplant and the reasons for these hospitalizations, number of days between registration on the transplant list and the transplant, number of hospitalization days during the transplant, surgical revisions, and number of hospitalization days after the transplant and the reasons for these hospitalizations.

#### Accompanying Patient Logbook

All accompanying patients participating in the study will document each of the meetings carried out with the patient in a logbook that will be available to all researchers and medical personnel in the project as well as in the REDCap application. In the logbook, the accompanying patient will provide information about the patient (last name and first name), the context of the meeting (health care facility, date of the meeting, start and end time of the meeting, person who requested the meeting, meeting number, and people present during the meeting), the stage of the patient’s trajectory (pre-, peri-, or posttransplant stage), meeting method (in person or remotely and location of the meeting), the themes addressed (general aspects, organizational aspects, consequences on daily and family life, and clinical aspects), the accompanying patient’s perceptions of the contribution to the patient and of the relationship with the patient, the difficulties encountered by the accompanying patient, unanswered questions, feedback to the clinical team and planned follow-ups, and any other comments deemed relevant by the accompanying patient. The accompanying patient’s logbook can be found in Multimedia Appendix 1.

#### Coordinator Logbook

The coordinator logbook will capture qualitative and feasibility data that include entries on the following: (1) questions or challenges reported by the participants during consent and baseline educational meetings; (2) details about participant recruitment and fidelity throughout the intervention; (3) the recruitment rate (ie, the proportion of contacted eligible individuals who are included in the study); and (4) the retention rate, defined as the proportion of included participants who are retained over the full follow-up period. Fidelity, or the degree to which the intervention was implemented as intended, will be measured as the proportion of included patients who complete their daily self-assessments over the full follow-up period. The reasons for each of these activities will also be categorized and described based on the detailed information recorded in the coordinator logbook.

#### Interviews and Focus Groups

All qualitative interviews and focus groups will be conducted individually, preferably in person and, if not, via videoconference or phone. Each interview and focus group will be recorded and follow a semistructured guide with open-ended questions and specific prompts. Although adapted to each stakeholder group, the interview guide includes broad questions on the individual’s experience of and thoughts on using the digital platform follow-up, with prompts on challenges and facilitators [38] as well as open questions to identify information and education needs during the study. The interview guides for each stakeholder group can be found in Multimedia Appendix 2.

With the agreement of patients, accompanying patients, HCPs, managers, and decision makers, all interviews and focus groups will be recorded and transcribed to ensure the reliability of the information collected. The recordings will be transcribed and uploaded into the QDA Miner software (Provalis Research) for further analysis and to identify expected and emerging themes.

### Data Analysis

All statistical analyses will be conducted using the R software (R Foundation for Statistical Computing) [39]. For the accompanying patient and coordinator logbooks, descriptive statistics (medians, means, and SDs) will be calculated. Economic analysis will be conducted using the Stata software (StataCorp).

Concerning the various questionnaires, descriptive statistics (medians, means, and SDs), chi-square tests, and 2-tailed *t* tests will be calculated on the populations studied for the sociodemographic characteristics of the patients (eg, age, sex, and characteristics of the care pathway) as well as on all the data obtained at each measurement time (ie, at T0 [assessment to be on the transplant waiting list] and then at 3, 6, 9, and 12 months). Medians, means, and SDs will be calculated for all patients.

For individual nutritional and physical conditioning optimization plans, an analysis will be conducted of the gaps between what was proposed and what was achieved. Statistical analyses will also be used to compare the patient’s ability to follow the plan before and after the LT. In addition, multivariate analyses will be conducted based on the intensity of the support provided by the accompanying patients.

For qualitative data, a thematic analysis will be conducted. Coding will be carried out independently by a research agent, students, and the researchers to (1) identify points of convergence and divergence in what is said by the different stakeholders, (2) codify the data using the QDA Miner software to systematize the analysis, (3) build emerging themes, and (4) highlight the convergences and divergences of the intervention.

The quantitative and qualitative data will be compared and analyzed to improve our understanding of the intervention [18]. The barriers and facilitators identified through semistructured interviews with the stakeholders (objective 2) will be used to interpret the findings on patient outcomes, the implementation, and the health services (objectives 1, 3, and 4).

### Specific Analysis for Each Objective

#### Objective 1: To Quantitatively Evaluate the Implementation Outcomes of a Patient Platform With Connected Devices and Accompaniment by a Former Patient

Implementation outcomes will be summarized using descriptive statistics. Acceptability and usability will be evaluated using a linear mixed model for each corresponding outcome. The evaluation of perceived response burden and fidelity will be conducted using a generalized linear mixed model for each corresponding outcome, which is appropriate when the dependent variable is not continuous. For all these calculations, the dependent variable of each model will be the outcome, and the independent variables will be the different time points of data collection (Table 3), 3 sociodemographic variables that are reported to influence patient portal use (gender [man or woman], age [≤50 or ≥50 years], and ethnicity), clinical data from the Stroop test, and the presence or absence of relatives during the transplantation process. The goals of testing each model are to determine whether the outcome’s mean score changes significantly over time and whether it differs significantly between the groups represented by the sociodemographic variables over time.

Thus, for acceptability and usability, if at each time point the outcome’s mean score is greater than or equal to the predefined success threshold, then the target will be considered met. If it is below the threshold, we will use a unilateral *t* test to test the null hypothesis of threshold attainment as being slightly below this mark does not imply failure given the sample mean’s variability. To evaluate threshold attainment during the evaluation of perceived response burden and fidelity, if the observed proportion is under the predefined success threshold, a unilateral *z* test will be conducted as it is appropriate for hypothesis testing with proportions. Finally, an evaluation of feasibility will be conducted by comparing the observed recruitment and retention rates with the predefined success thresholds at the end of the recruitment period and at the 1-year patient follow-up, respectively. If the observed rates are greater than or equal to the success threshold, the target will be considered met. If they are under the success threshold, we will use a unilateral *z* test to test the null hypothesis of threshold attainment.

#### Objective 2: To Identify Implementation Barriers and Facilitators Based on Semistructured Interviews With Stakeholders

To identify implementation facilitators and barriers and their proposed solutions, 2 analysts will conduct an analysis of the content extracted during the study. The source materials will include semistructured interview and focus group transcripts with the HCPs and the accompanying patients’ logbook entries. The analysis will be conducted in three phases [40]: (1) the preparation phase, or the period during which the analysts become familiarized with the data set; (2) the organization phase, when the analysts systematically code the data using the QDA Miner software to identify implementation facilitators and barriers while remaining open to any possible emerging categories; and (3) the reporting phase, which consists of presenting and discussing the identified categories during monthly team meetings with the clinical and research teams to identify discrepancies either in coding or interpretation, ensuring data reliability.

#### Objective 3: To Describe Service Outcomes in Terms of Health Outcomes and the Rates and Nature of Contact With the Accompanying Patient

A qualitative analysis will be conducted of the patients’ health status, including visits to emergency rooms and continuous clinical monitoring as well as the reasons underlying participant contact with the team of HCPs (Table 1). This information will be monitored continuously and extracted from the medical records. Moreover, a content analysis will be conducted to assess the nature of contact with the accompanying patient based on the data collected in the accompanying patient logbooks. In addition, a content analysis will be conducted to assess expectations and experience with the health care model as well as the user experience with the digital platform and COs using data collected during interviews and from discussion groups (Multimedia Appendix 1).

#### Objective 4: To Describe Patient Outcomes Based on the Daily Self-Reported Health Data, Including Symptoms, Use of Health Services Beyond the Intervention, and Patient Satisfaction With Teleconsultations if Received

Descriptive statistics will be used to report patient outcomes collected from medical records (Table 1), daily health data self-reported through the use of COs, self-reported questionnaires completed throughout the study (Table 3), and patient satisfaction with teleconsultations and tools. For the analysis, each patient constitutes their own reference and comparison. Thus, δ calculations will be conducted, derived by comparing individual questionnaire results with outcomes from the same questionnaires at other time points throughout the study (ie, baseline vs time points before and after transplantation). For continuous outcomes, the mean, SD, minimum, and maximum values will be reported. For ordinal and nominal qualitative outcomes, absolute and relative frequencies (proportions) will be reported. Descriptive statistics will also be presented by gender and age group.

#### Objective 5: To Assess the Cost-Effectiveness of the Intervention

A cost minimization analysis will be used to meet this objective. The out-of-pocket costs for the patient and the caregiver will be measured using the cost for patient questionnaire, which was developed and validated in Quebec [41,42]. The costs for the health care institution will be measured using the PowerHealth software (PowerHealth Solutions) used at the CHUM, which combines financial data with data in patient clinical files. The costs for the health care system will include the costs borne by the institution, including physician remuneration. To identify these costs, a questionnaire on medical visits and consultations will be sent to the patient every 3 months so that the patient can indicate the number and nature of these events. The *Régie de l’assurance maladie du Québec* pricing manual will then be used to identify these costs.

### Ethical Considerations

#### Approval and Consent

Ethics approval was obtained from the research ethics board at the Centre hospitalier de l’Université de Montréal (CHUM), Quebec, Canada (study ID: 20-5185). The research team and principal investigators at the hospital are responsible for recruitment and monitoring of participants. Any major modifications or protocol deviations are discussed with the other principal investigators during monthly meetings, and any major protocol modifications are reported and submitted to the ethics board for approval. Participants will be approached by the project medical team and then by the research team to inform them about the objectives, benefits, and risks of the research. If the participant is accepted for LT, the research team will meet with the patient and have them sign the consent form.

#### Safety and Anticipated Risks

There are no direct risks to participants in this study. Data security risks will be addressed through numerous measures, such as copying data to the CHUM Research Centre (CRCHUM) server and protecting them with a user code and password. Moreover, the study questionnaires, semistructured interviews, and accompanying patient logbook sessions may lead to distress related to the transplant procedure or follow-up, the economic details collected, or depression and anxiety explorations. Therefore, these research tasks carry a risk of emotional vulnerability. Individuals who experience psychological distress because of their involvement in the study will be instructed to inform a member of the study staff. Regular psychological assessments or support services will be provided by the team’s psychologist throughout the study.

#### Confidentiality, Data Management, and Cybersecurity

All collected data and personal information will be deidentified (coded) and kept in computer format at the CRCHUM. No identification of individual participants in any images of the manuscripts or supplementary materials will be possible. When the study results are released, participants will not be identifiable. The project’s principal investigator (MPP) and coinvestigators will have access to the data and study results. All computer data will be copied to the CRCHUM server and protected by a user code and password. Research data will be retained for 10 years after project closure. The person in charge will be the principal investigator of the study, MPP. To join the scientific community, peer-reviewed scientific publications will be recommended.

#### Compensation

No financial compensation is offered for participating in this study. However, patients will have access at no cost to all COs and follow-up consultations.

## Results

Several study-related activities have already been carried out from July 2022 to October 2023 concerning the intervention implementation, research, and the platform promoter, in addition to funding. Some steps are ongoing.

### Implementation Status

To complete the GRIT-F clinical team and strengthen the capacity to monitor patients at the pre-, peri-, and posttransplant stage, a nutritionist, a nurse, a kinesiologist, and a psychologist have been recruited. A total of 5 accompanying patients have been recruited and, along with the clinical team, trained on their roles and how to work together. A certain number of working committees have also been set up, including the following:

1. A steering committee comprising the physician responsible for the transplant clinic, the nursing comanager, the principal researcher, the 2 research assistants, and 3 accompanying patients. This committee meets every week to oversee the implementation of the project.
2. An education committee was also created in September 2023 to develop the tools needed to carry out the educational assessment, including identification of the patient’s LT pathway, and the skills that patients and relatives can develop throughout the LT pathway. This committee also helps identify the resources that can be mobilized to support patients and their relatives and integrate these resources into the life course of the patients on the digital platform. This committee consists of not only the 5 accompanying patients but also all the members of the clinical team, the principal investigator, and representatives of the digital platform, as well as specialists in therapeutic education and health literacy. The committee meets every 15 days.
3. A clinical-technological committee ensures that the development of the platform is aligned with the clinical context and patient experience. This committee comprises the clinical team, accompanying patients, and digital platform representatives. It meets every 2 weeks.
4. A committee on prescriptions and nursing roles has also been set up to develop collective prescriptions to give nurses greater flexibility in their activities.
5. Finally, an accompanying patient community of practice has been formed, bringing together the 5 accompanying patients each week to discuss these practices and react to the research tools intended for them.

### Research Status

In total, 2 research assistants and a PhD student have been recruited for ethics monitoring, development of the data collection plan and collection tools, recruitment of participants, and analysis of the data, as well as to ensure adherence to deadlines. This study will be conducted in a tertiary hospital. Written informed consent will be obtained from all participants before any study activities begin. Patient recruitment is expected to start in November 2023. All questionnaires; technological platforms; and clinicians, stakeholders, and accompanying patient personnel have been recruited for this study. The protocol was presented at the Canadian Association for Health Services and Policy Research conference in May 2023. In addition, the researchers will meet monthly to discuss the research plan, including the economic evaluation.

### Platform Status

A contract has been signed between a digital platform developer and the CHUM, and 2 members of the company have been specifically designated to the project. Personalized training was offered by the company to all members of the clinical team, the accompanying patients, and the research team.

### Funding Status

This study secured funding from the *Institut de la pertinence des actes médicaux* in June 2022.

## Discussion

### Expected Findings

Through this study, the CHUM LT clinic aims to explore the effects of a new health care model, called TAC, on the patient trajectory in LT. The project will be implemented in three phases: (1) a rapid investigation window model in pretransplant assessment will be developed with the introduction of COs and the mobilization of a kinesiologist, a nutritionist, a pharmacist, 2 nurses, a psychologist, a social worker, a computer technician, and accompanying patients; (2) the model will then be implemented throughout the care pathway, including after transplantation, through the integration of an digital platform that includes not only monitoring via COs but also access to educational material coconstructed with patients; and (3) data from the platform will be integrated into the electronic medical record and the CHUM computerized clinical file. Adjustments will also be made to the digital platform to ensure optimal follow-up with the COs as well as for access to personalized medication and nutritional and physical exercise treatment plans.

To our knowledge, this study will be the first to investigate and attempt a reorganization of the care pathway in LTs by optimizing trips to the hospital, reducing the time required for the patient to be registered on the transplant list, engaging an interdisciplinary team to cocreate strategies that encourage patient commitment to their own health promotion, mobilizing each health professional in a relevant manner, and establishing the use of COs to monitor health progression as well as the use of accompanying patients who have already undergone a transplant at the CHUM. Although many studies have brought to light the key roles played by patient monitoring throughout an intervention [43-45] and the inclusion of educational programs provided by HCPs to patients [46-48] in intervention effectiveness over time, this is the first study to integrate both components while also involving an interdisciplinary team to support and explore their application by combining them into a single health care model. The originality of this study lies in the fact that it integrates accompanying patients as full-fledged members of the clinical LT team to assess the potential effects at the clinical and organizational levels, as well as integrating them into the process of creating educational programs for the patients. This study introduces the use of multiple COs, which could have a major impact on patient plans by closely monitoring the patient’s health status. This may lead to modifications in personalized nutritional and physical exercise treatment plans that will improve recovery rates after LTs. Moreover, the mixed methods nature of this study allows us to explore the variability between settings to delineate various clinical scenarios and document patients’ economic, emotional, and personal realities during the transplant process.

If this new health care model is relevant, it might maintain or improve the clinical condition of patients before and after all types of transplantation through a more personalized and closely monitored follow-up, allowing for the creation of a single platform adapted to several users and cases. The model would also optimize the use of medical resources throughout the continuum of the LT process as well as reduce treatment costs for patients, the health care facility, and the health system.

### Limitations and Challenges of This Research

This study, as complex and multidisciplinary research, is not without its limitations and challenges. Some of these challenges and solutions are, first, the management of a large interdisciplinary team and the standardization of peer support. To be able to overcome any difficulties related to the cohesion of the team, regular committee meetings will be held once a month to inform the HCPs, researchers, stakeholders, and accompanying patients about progress in the study; redirect guidelines; and discuss participant’s cases to achieve a better cohesion among the members of the interdisciplinary team. Another limitation is the large number of questionnaires in addition to the introduction of COs (which increases the difficulty of training and use) as it may increase the burden on patients, which may lead to compliance issues. Some ways to reduce this workload are to select the questionnaires that are strictly necessary at each point during the study and use simpler versions of the questionnaires that require more time to complete. Participants will be informed beforehand of their consent to the tasks and the burden that this might carry. Moreover, the digital platform and CO provider will continuously monitor any difficulty using the COs, providing support and maintenance at all times, thus decreasing the burden on the patient.

Barriers to the generalization of research findings also need to be considered. This study is implemented by capturing data from deconditioned patients and factors specific to the liver failure context. However, if the results of the study are positive, our analysis of the implementation outcomes will shed light on the best conditions for implementing the model in other transplant care trajectories within the CHUM (ie, kidney and lung transplantations).

Finally, as this study will collect a large quantity of data from various medical and nonmedical sources, the data management platform REDCap will be used to apply, manage, and integrate all data collected.

### Conclusions

By implementing this new health care model in the trajectory of LT recipients at the CHUM, it can be tested in an environment where the clinical team is motivated and united and where the number of patients is manageable. This new model has the potential to ensure that patients reach their transplantation in better health. This would ultimately reduce the average length of hospital stay and nursing unit care as well as promote better use of medical resources. In the event of positive results, this model could be transposed to all transplant units at the CHUM and across Quebec (potentially affecting 888 patients per year), but it could also be applied more widely to the monitoring of patients with chronic diseases. The lessons learned from this project will be shared with decision makers and serve as a model for other initiatives involving COs or digital platforms.

## Appendix

Multimedia Appendix 1 Accompanying patient logbook.

Multimedia Appendix 2 Qualitative interview guides for each of the stakeholder groups.

## Abbreviations

6MWT: 6-Minute Walk Test
CHUM: Centre hospitalier de l’Université de Montréal (University of Montréal Health Center)
CO: connected object
CONSORT: Consolidated Standards of Reporting Trials
CRCHUM: Centre hospitalier de l’Université de Montréal Research Centre
GRIT-F: *guichet rapide d’investigation en transplantation du foie* (rapid liver transplant assessment service)
HCP: health care professional
LT: liver transplantation
REDCap: Research Electronic Data Capture
TAC: Transplant’Action Connected in Liver Transplant

## Data Availability

The data sets generated during and/or analyzed during this study are available from the corresponding author (MPP) on reasonable request.
