# Supplementary figures and images for: Telehealth-Delivered Program and Accompanying Patients to Enhance the Clinical Condition of Patients Throughout a Liver Transplant: Protocol for a Mixed Methods Study
Source: JMIR Res Protoc. 2024 Mar 22;13:e54440. doi: 10.2196/54440 (PMC10998179; doi:10.2196/54440)

**
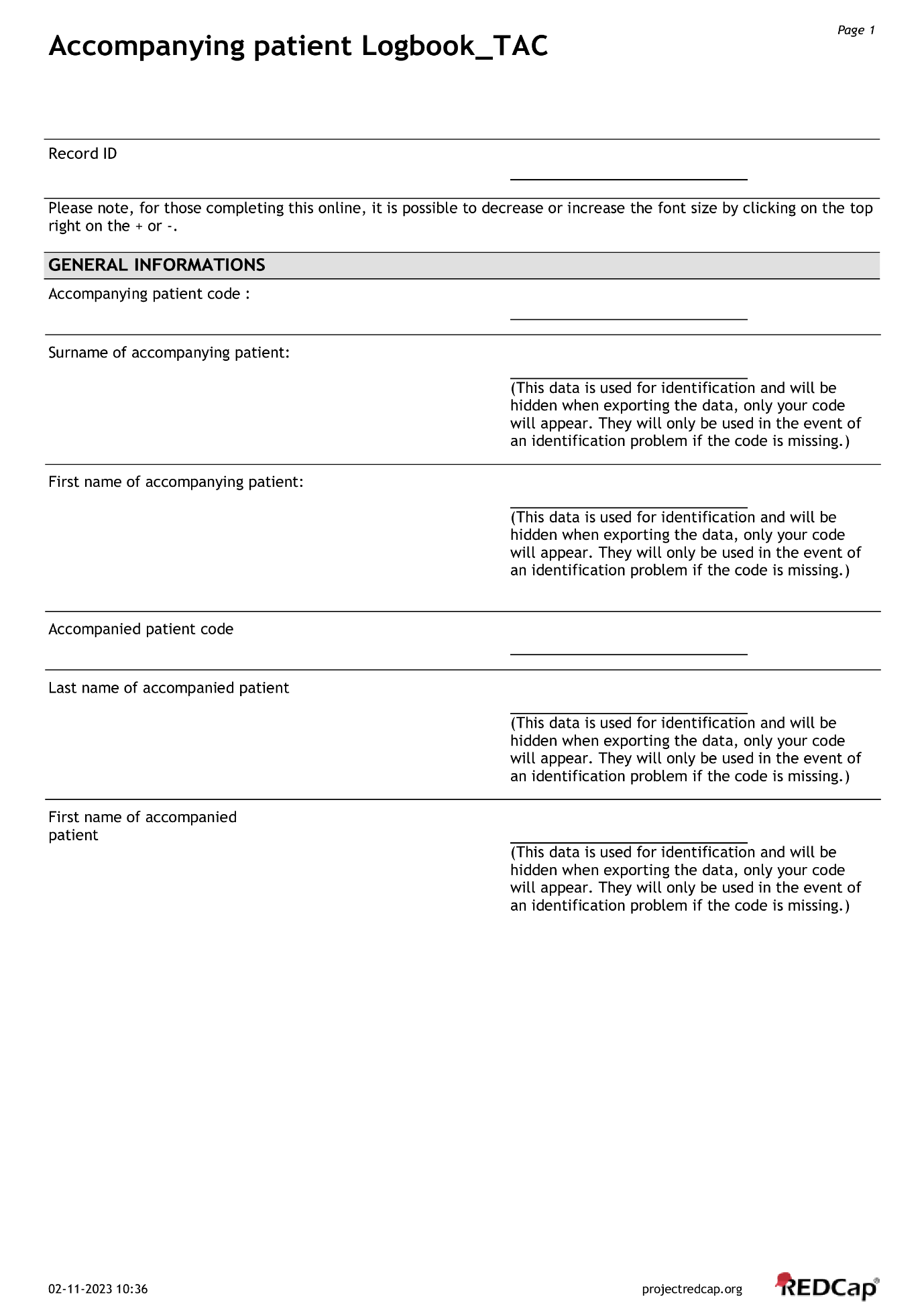

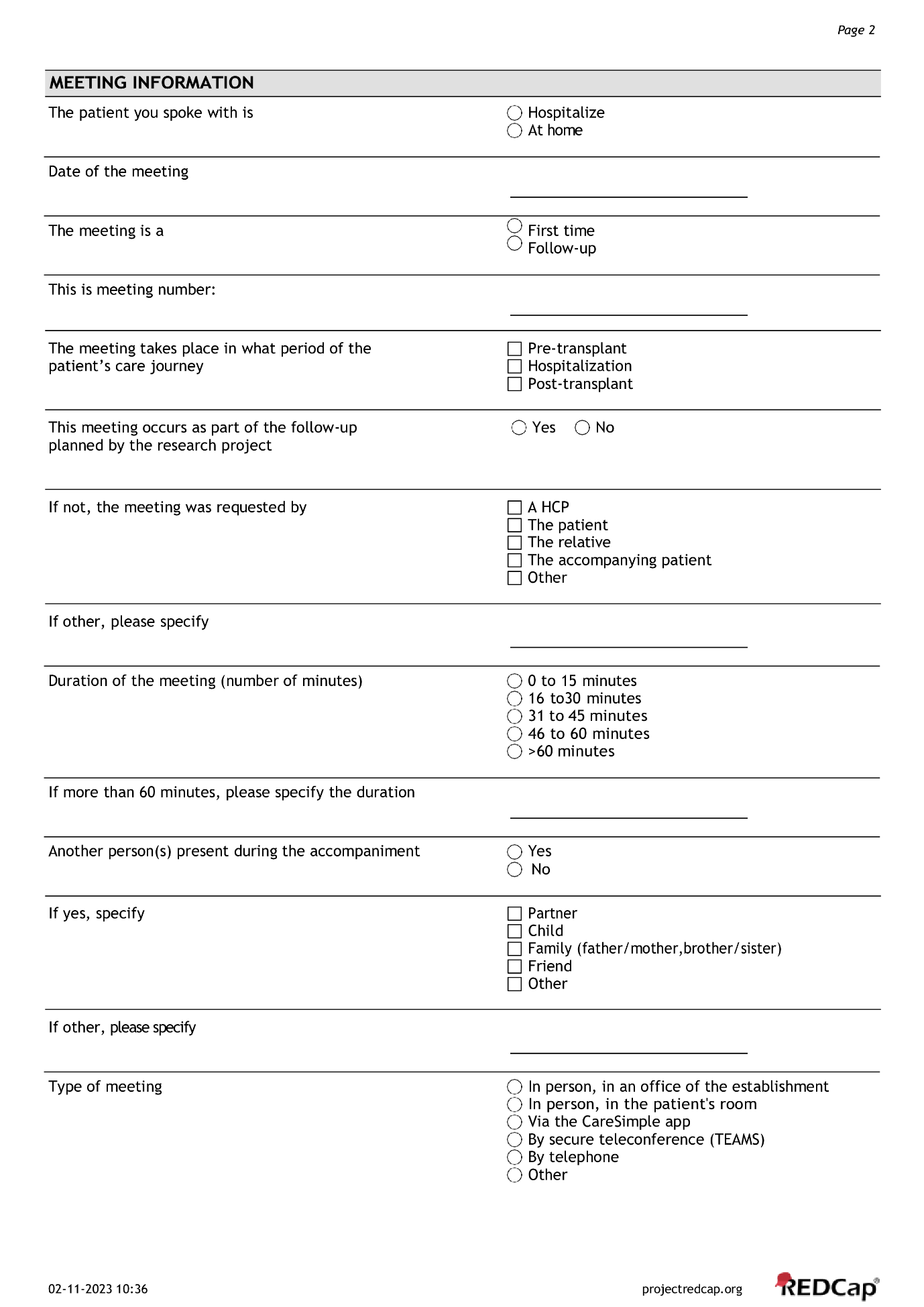

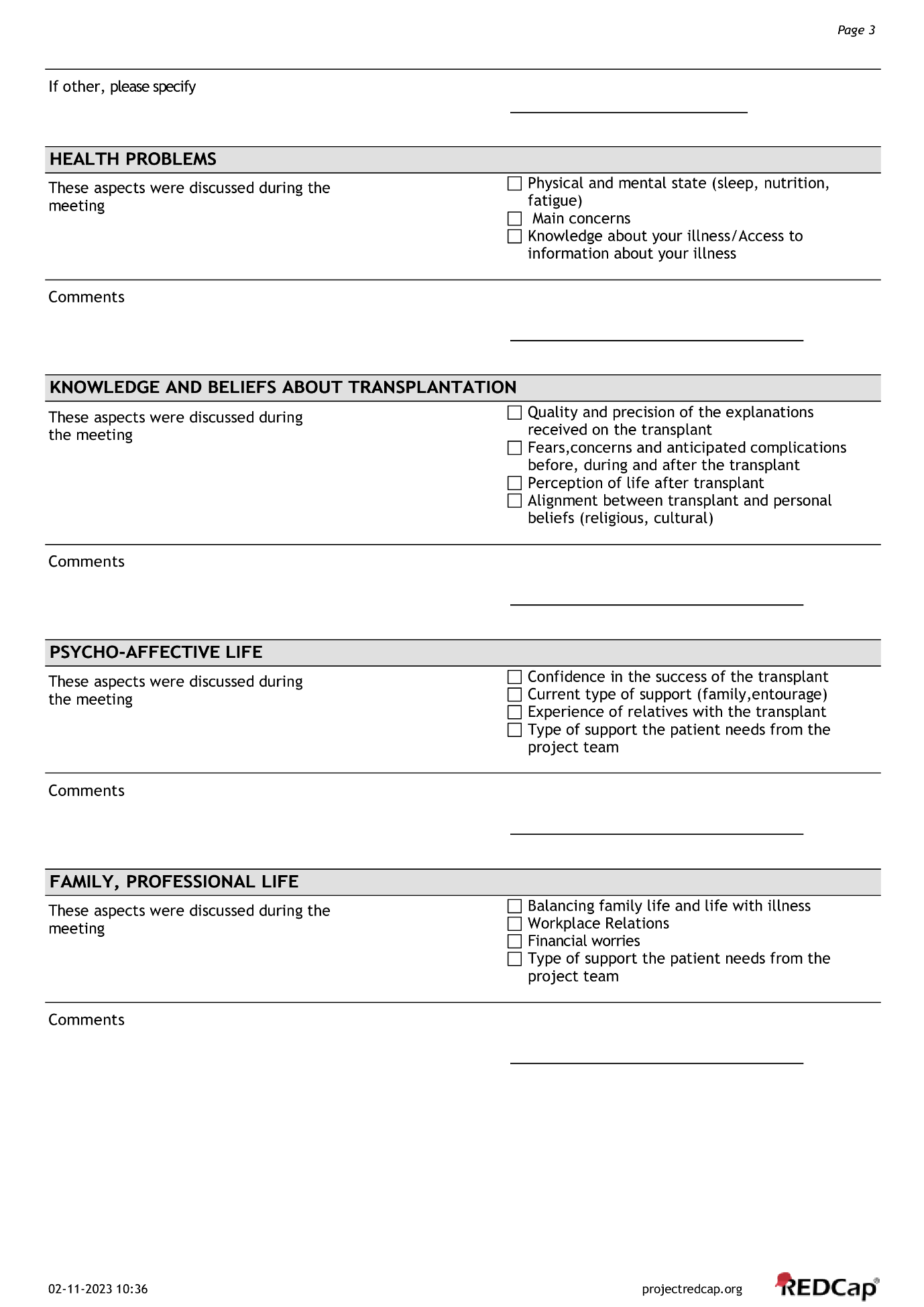

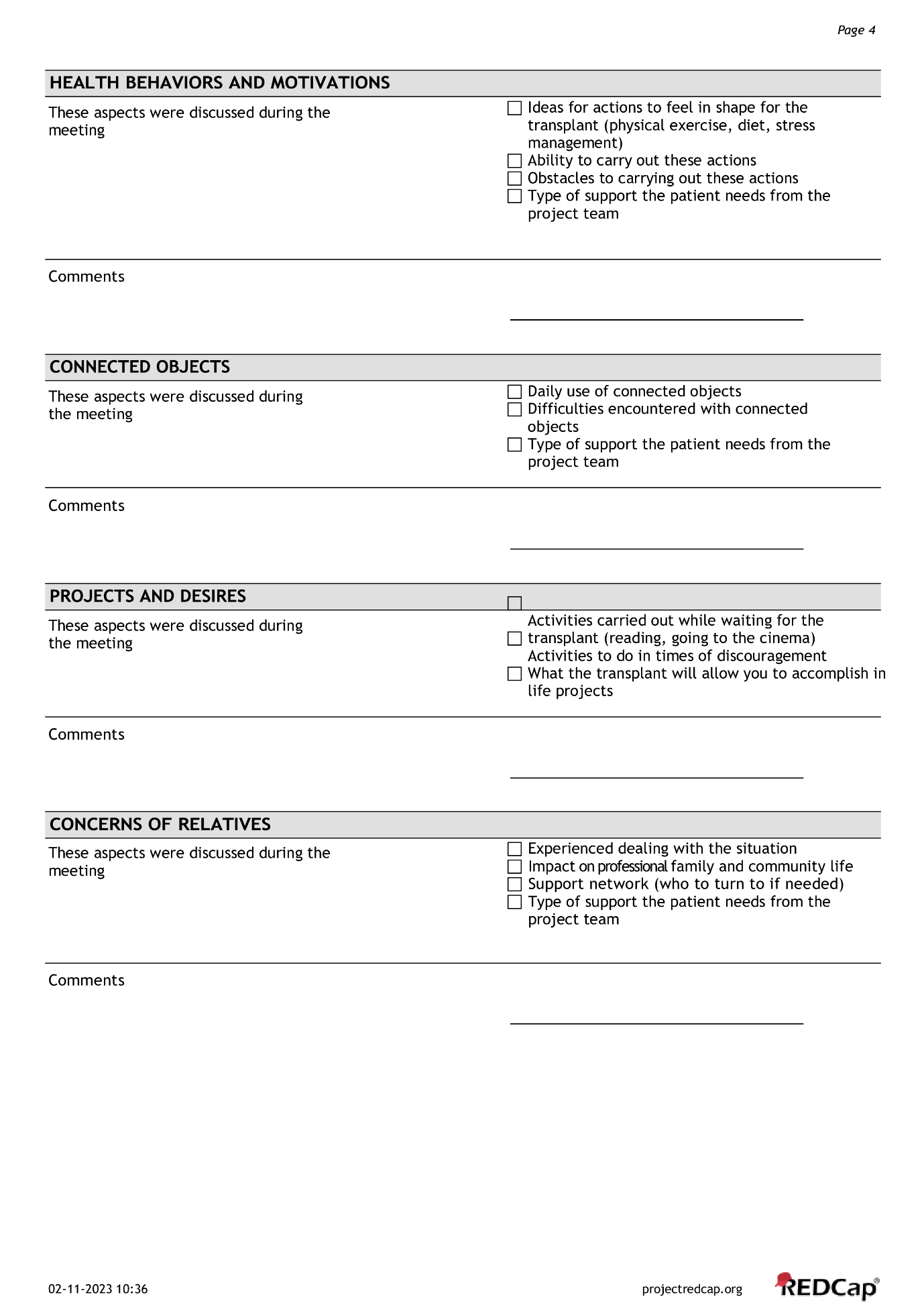

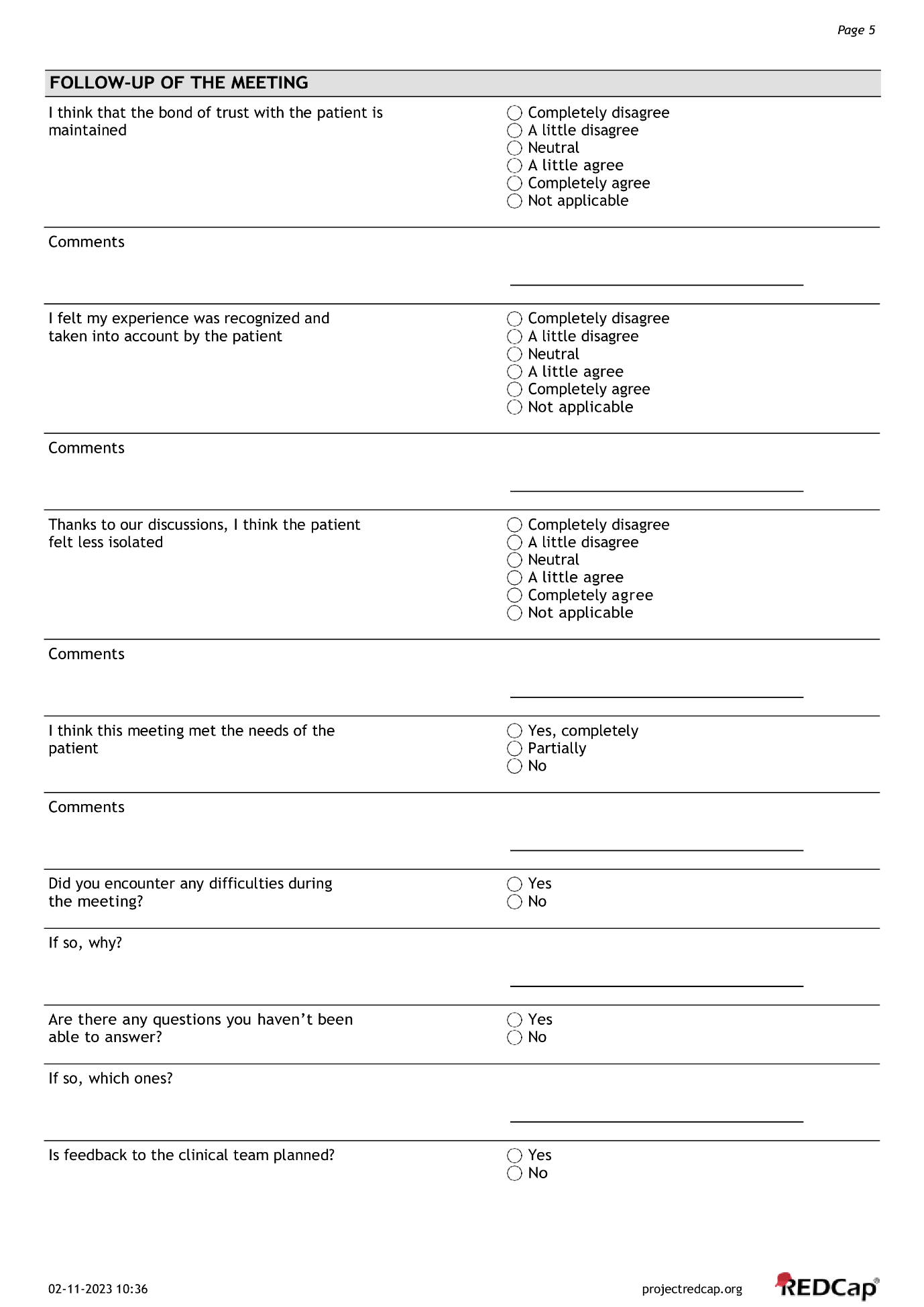

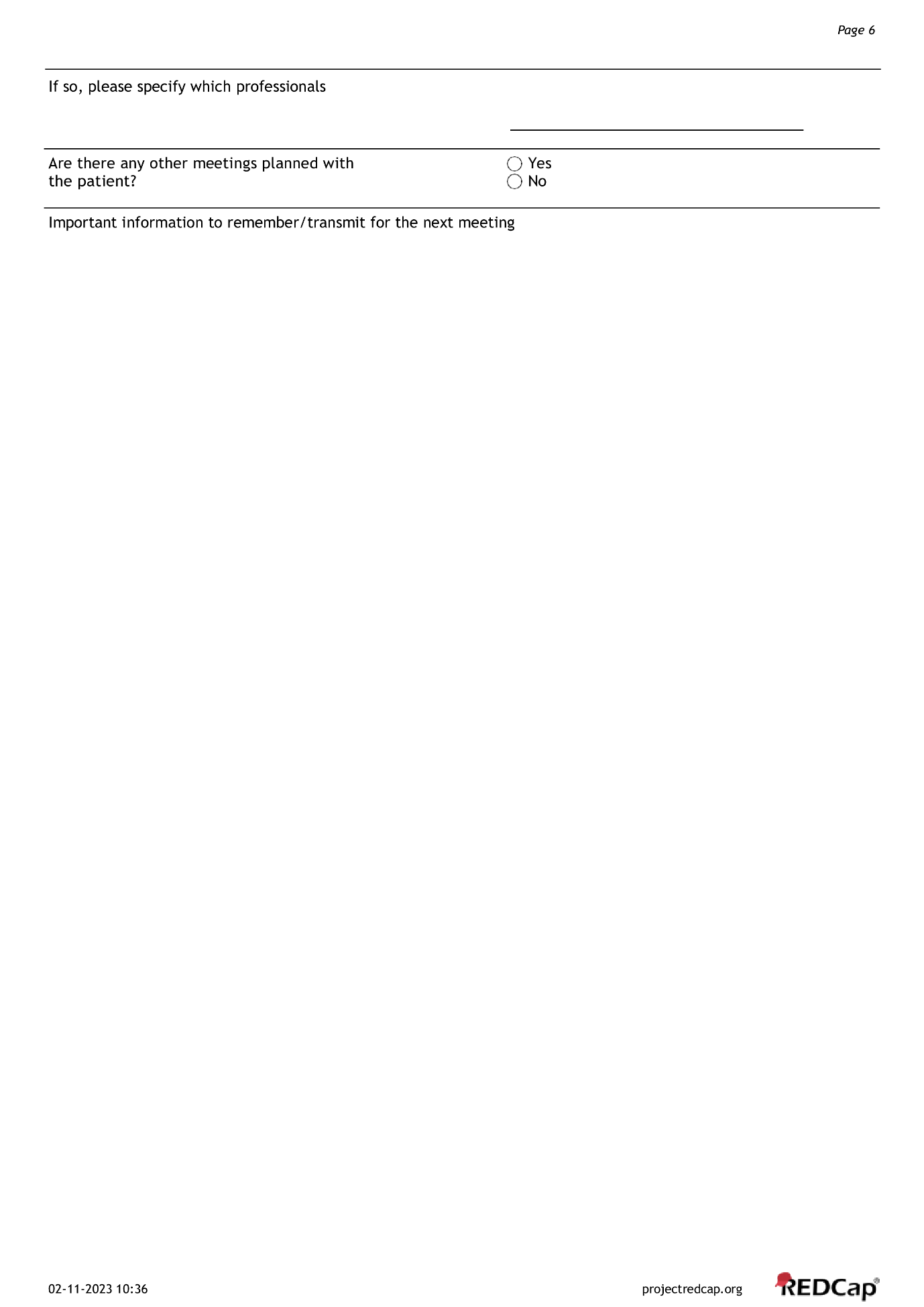
**

Supplement: Multimedia Appendix 1 [file resprot_v13i1e54440_app1.docx]
