# Supplementary material for: Telehealth-Delivered Program and Accompanying Patients to Enhance the Clinical Condition of Patients Throughout a Liver Transplant: Protocol for a Mixed Methods Study
Source: JMIR Res Protoc. 2024 Mar 22;13:e54440. doi: 10.2196/54440 (PMC10998179; doi:10.2196/54440)
Supplement: Multimedia Appendix 2 [file resprot_v13i1e54440_app2.docx]

***1.1 Interview / Focus Group: Patients***

***
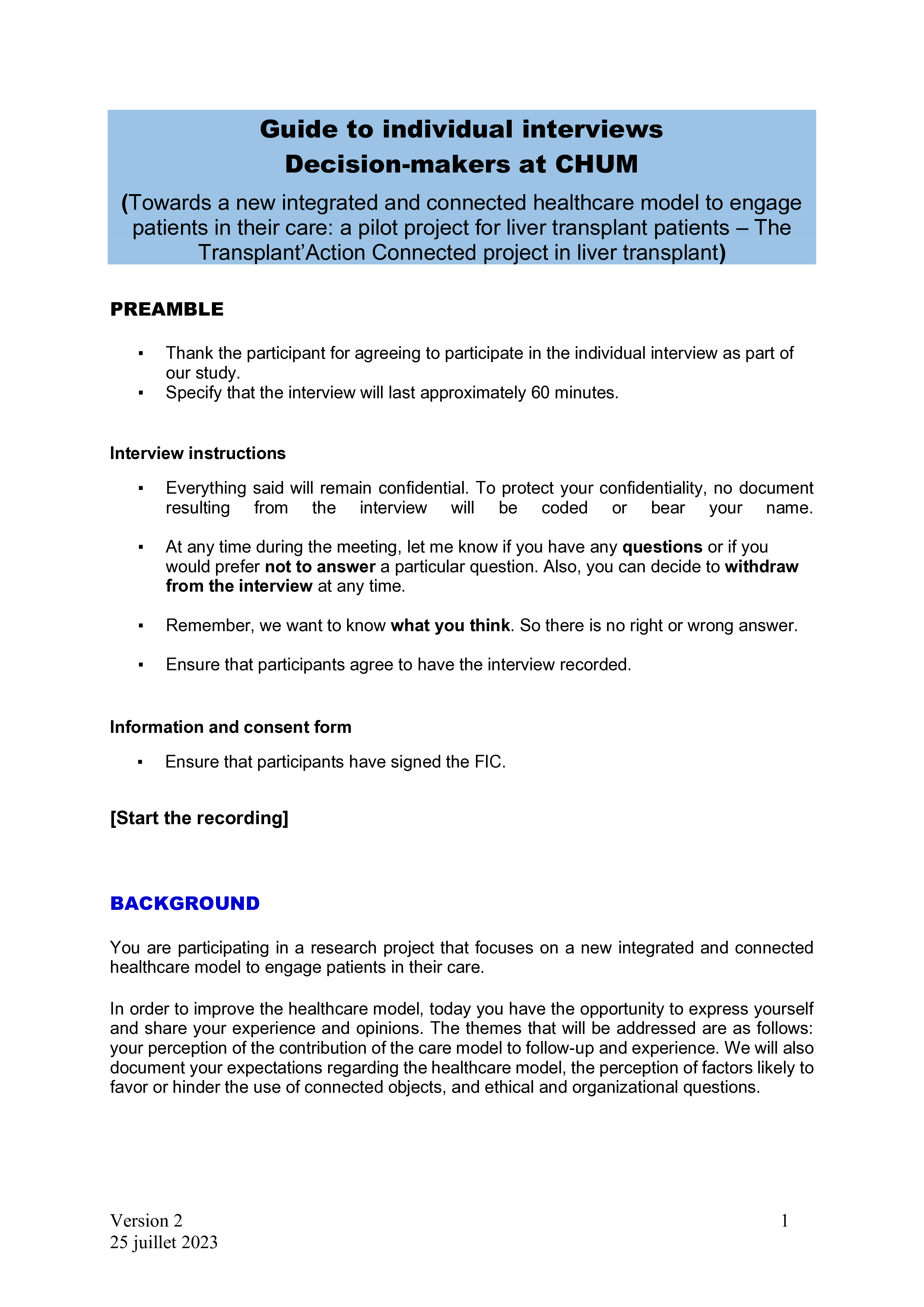

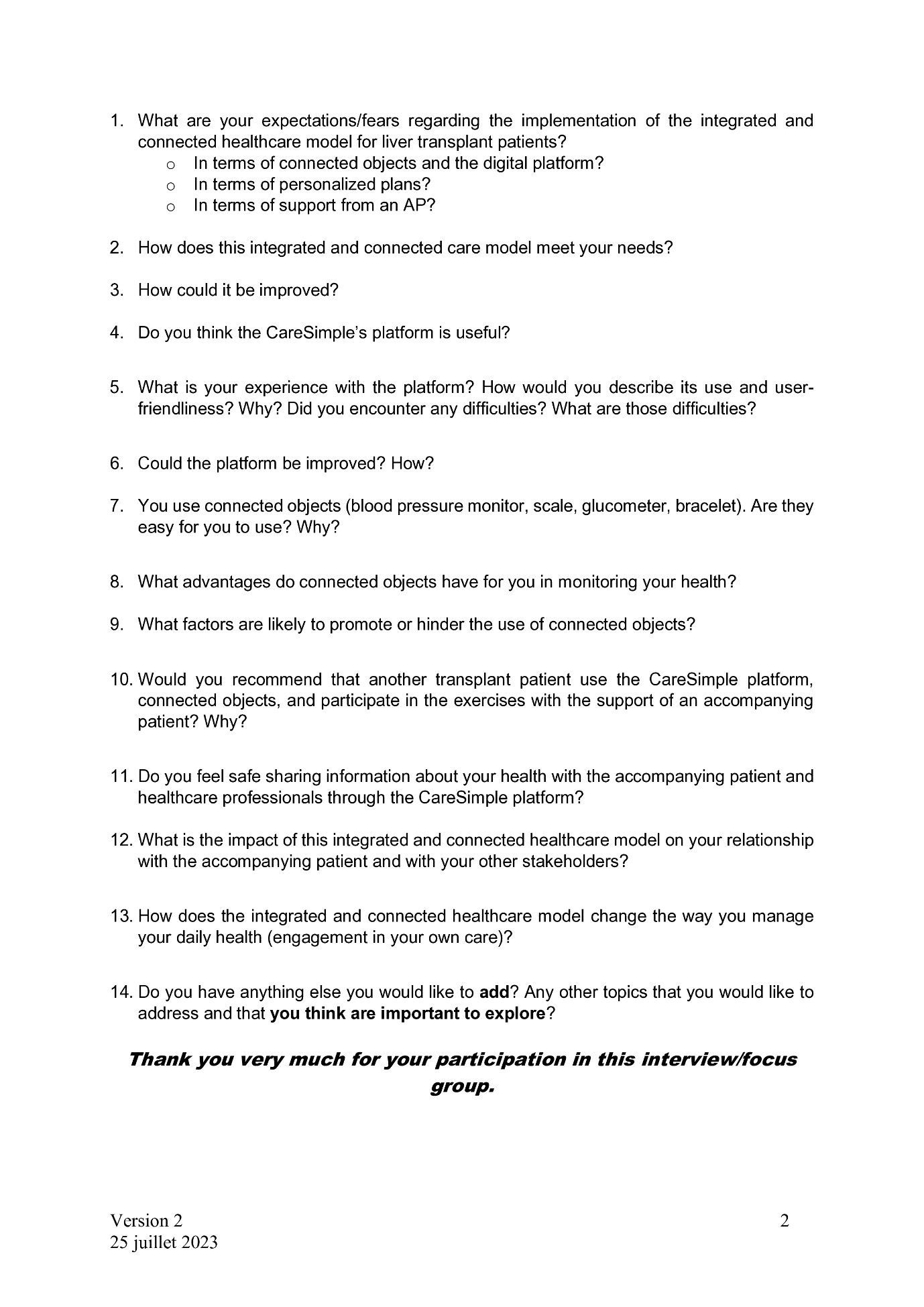
***

***1.2 Interview / Focus Group: Transplanted Patients***

**
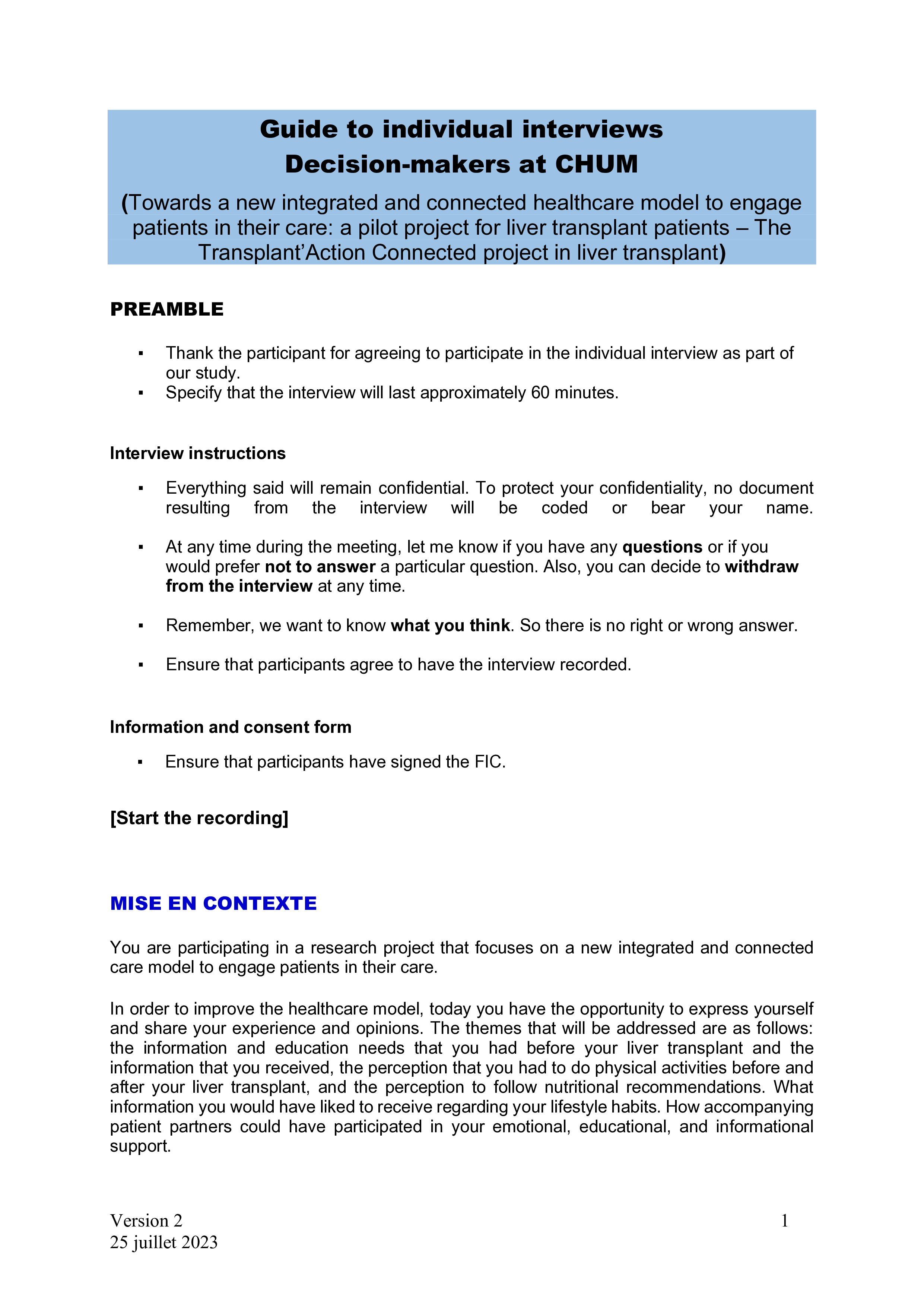

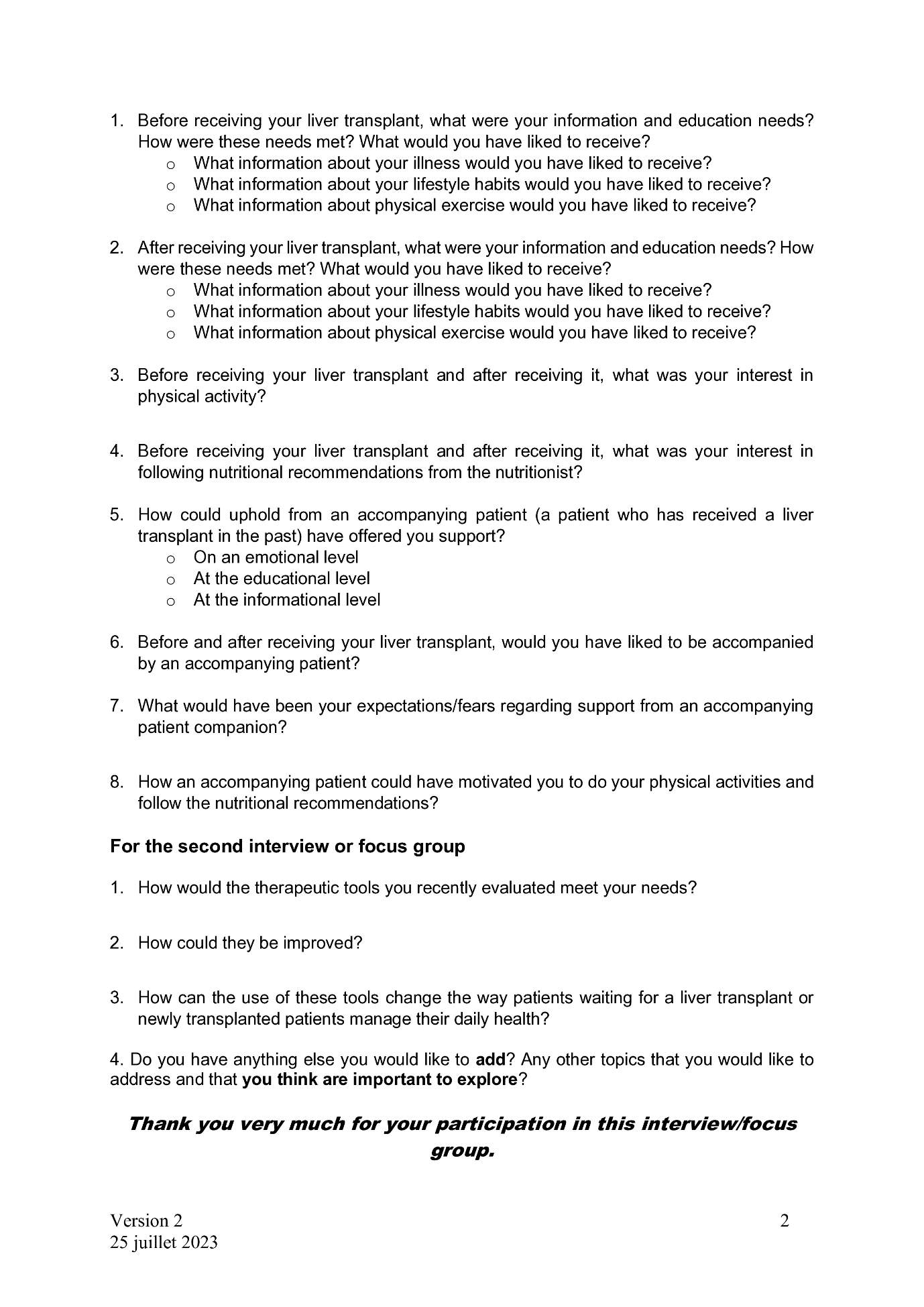
**

***1.3 Interview: Accompanying Patients (AP)***

***
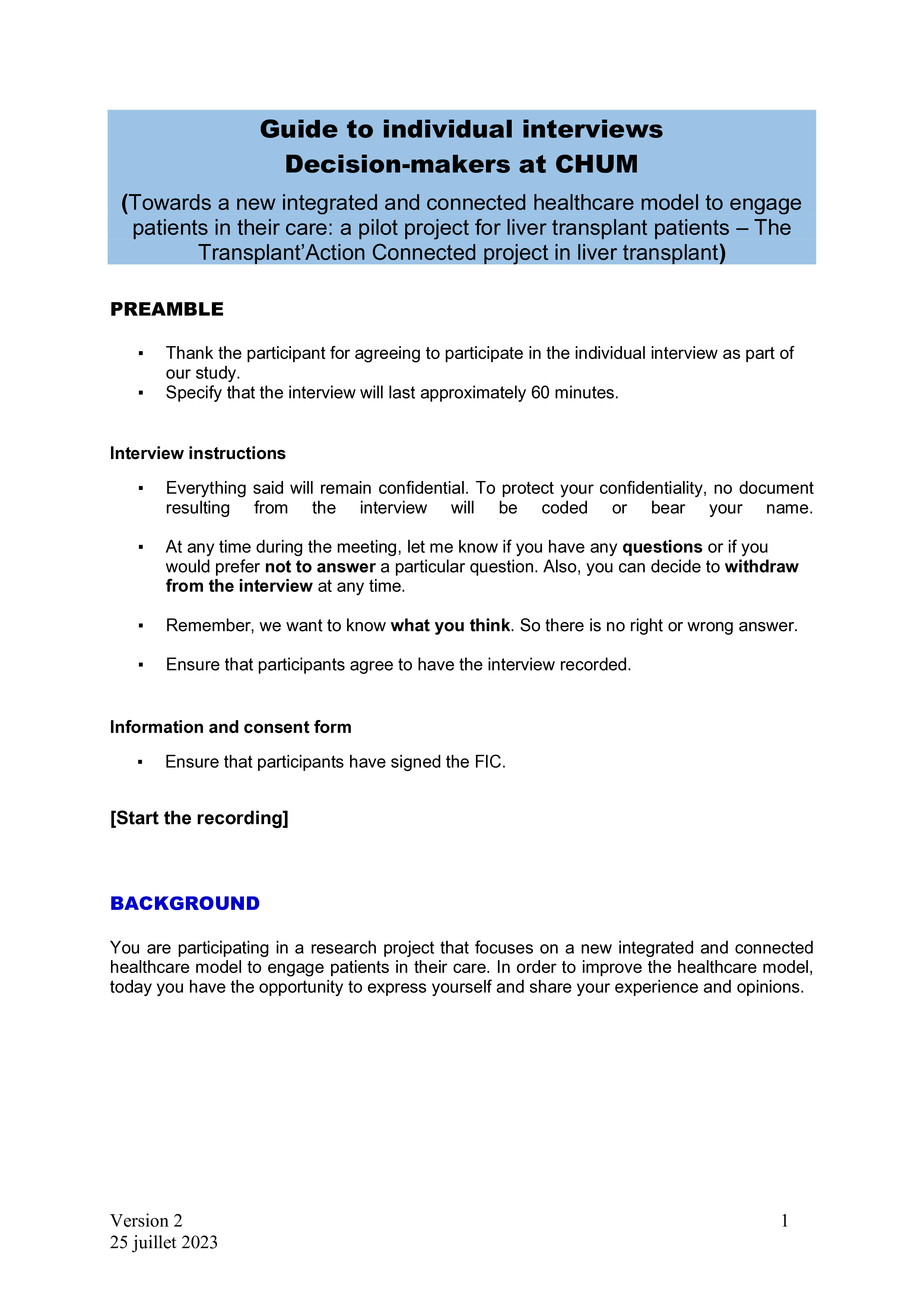

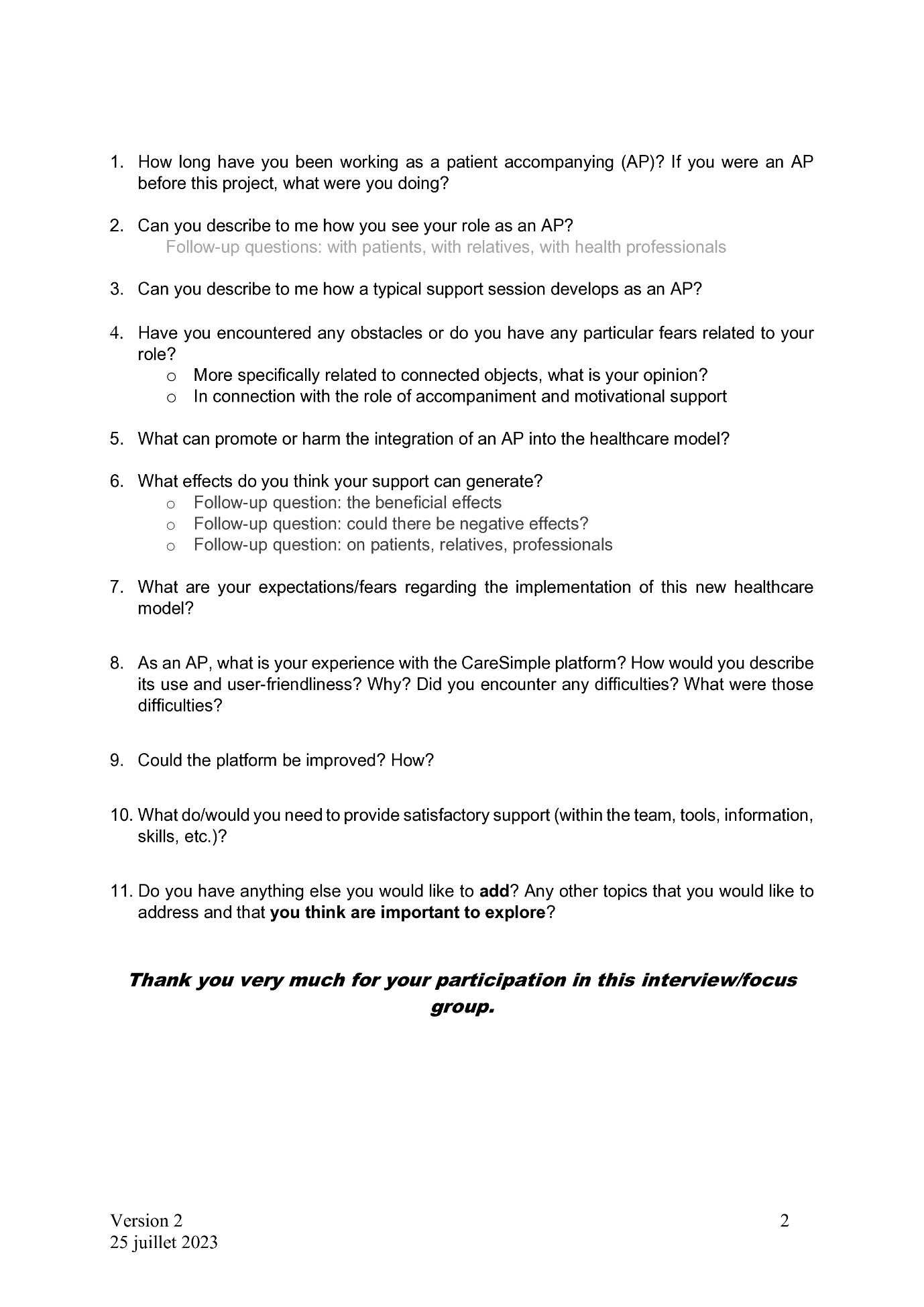
***

***1.4 Interview: Healthcare professionals***

***
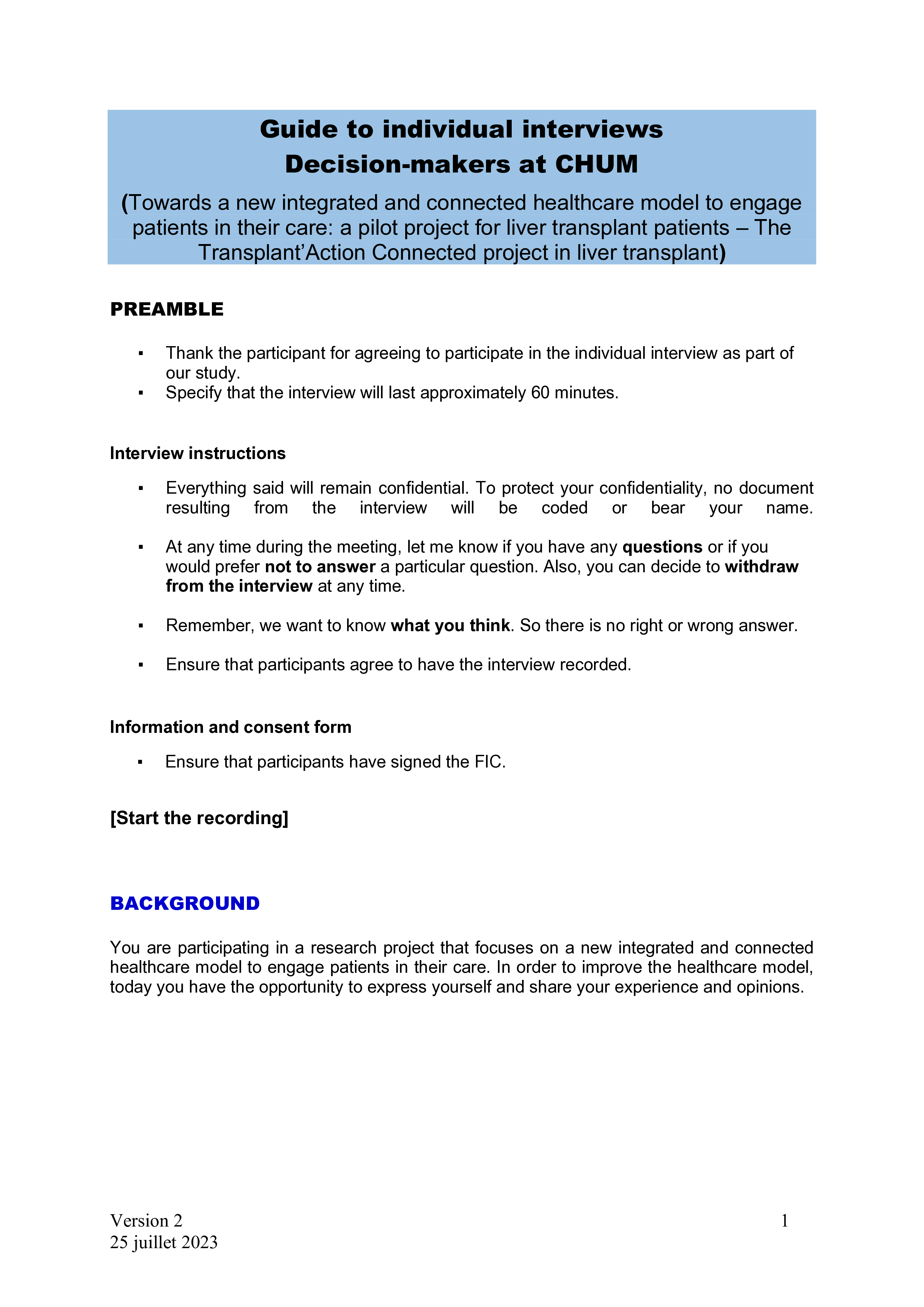

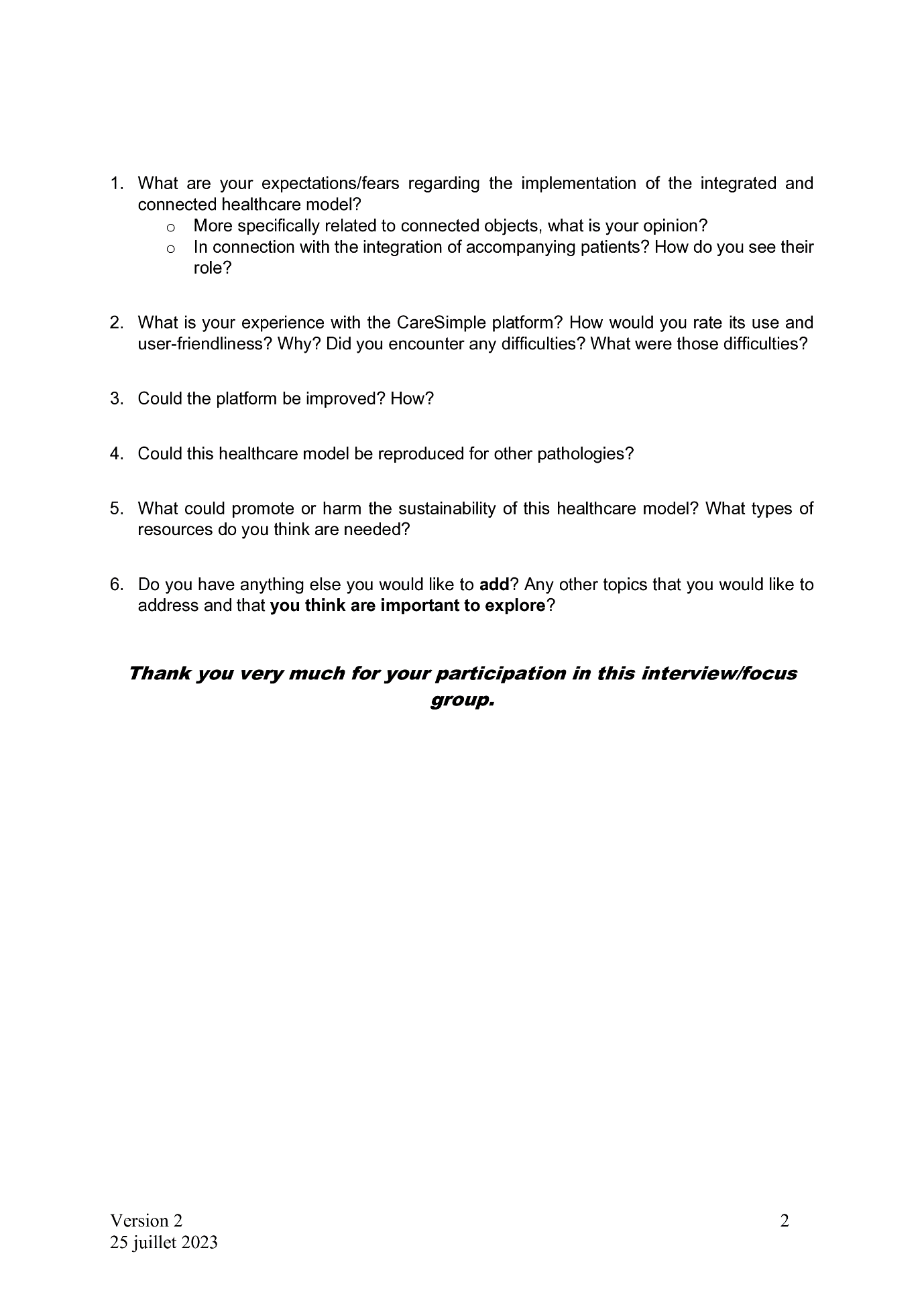
***

***1.5 Interview: Stakeholders***

***
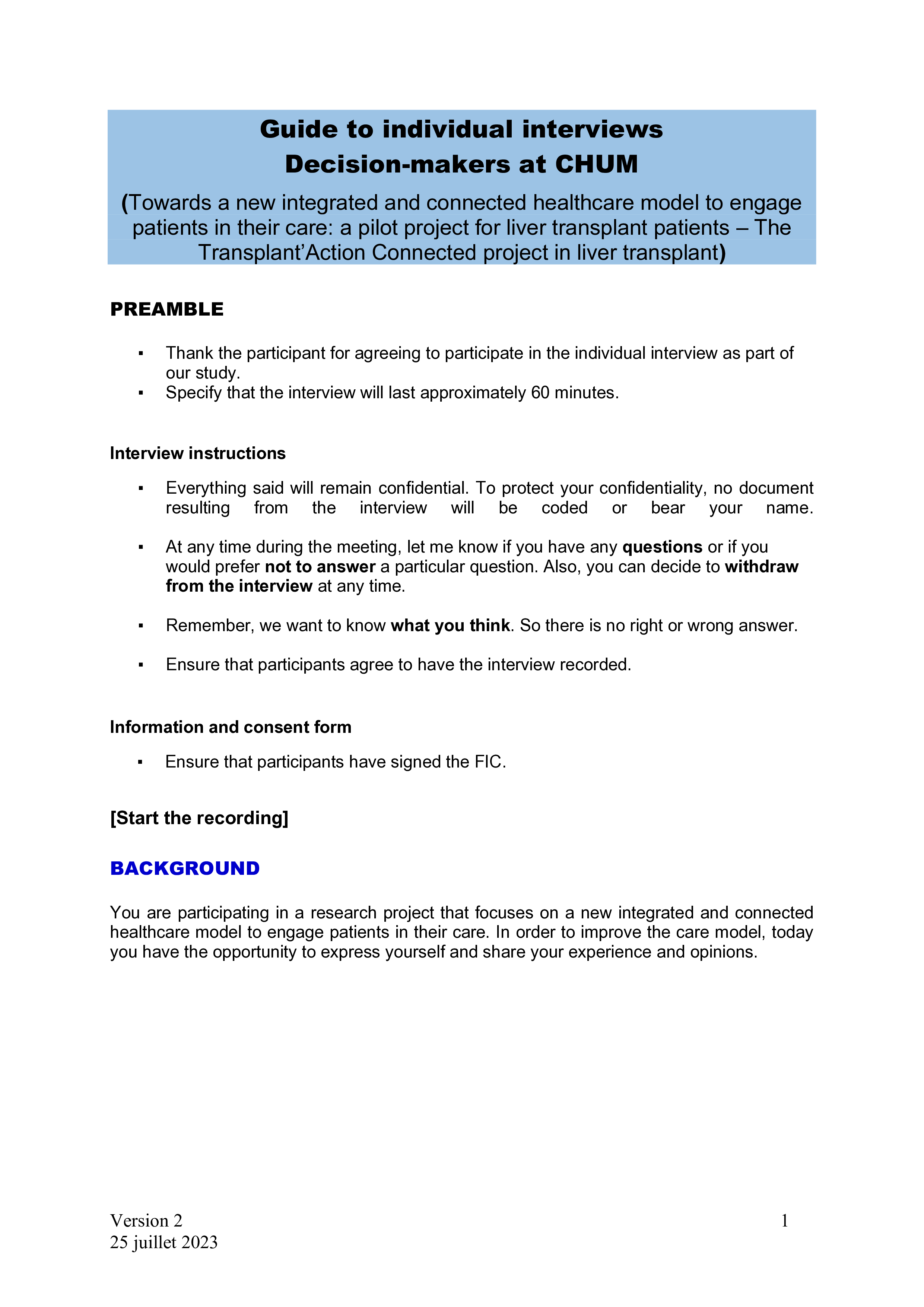

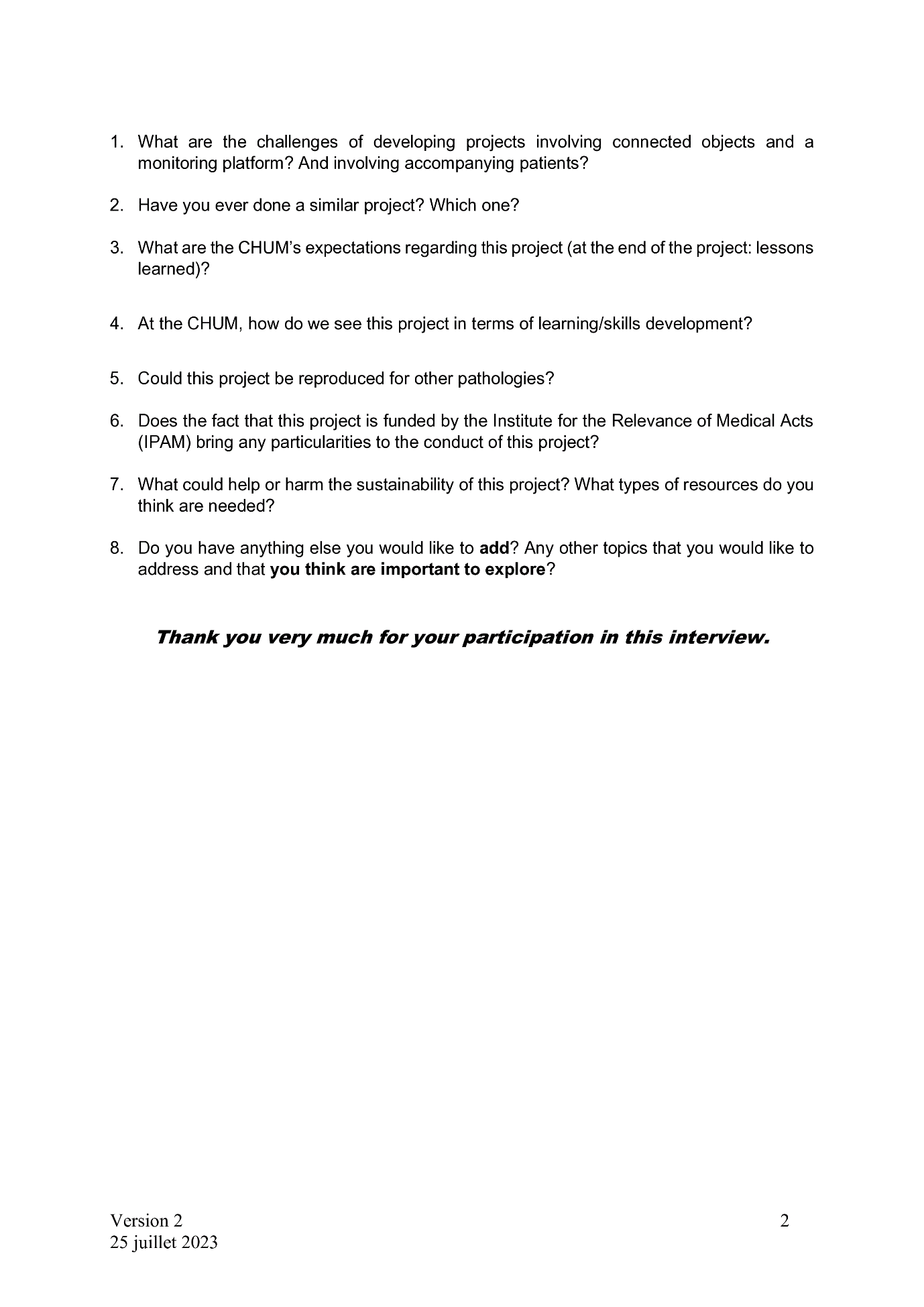
***
